# Information-Theoretical Analysis of a Transformer-Based Generative AI Model

**DOI:** 10.3390/e27060589

**Published:** 2025-05-31

**Authors:** Manas Deb, Tokunbo Ogunfunmi

**Affiliations:** Department of Electrical and Computer Engineering, Santa Clara University, Santa Clara, CA 95053, USA; togunfunmi@scu.edu

**Keywords:** machine learning, generative AI, transformer, information theory, mutual information estimation, information geometry, riemann manifold, fisher metric, geodesic

## Abstract

Large Language models have shown a remarkable ability to “converse” with humans in a natural language across myriad topics. Despite the proliferation of these models, a deep understanding of how they work under the hood remains elusive. The core of these Generative AI models is composed of layers of neural networks that employ the Transformer architecture. This architecture learns from large amounts of training data and creates new content in response to user input. In this study, we analyze the internals of the Transformer using Information Theory. To quantify the amount of information passing through a layer, we view it as an information transmission channel and compute the capacity of the channel. The highlight of our study is that, using Information-Theoretical tools, we develop techniques to visualize on an Information plane how the Transformer encodes the relationship between words in sentences while these words are projected into a high-dimensional vector space. We use Information Geometry to analyze the high-dimensional vectors in the Transformer layer and infer relationships between words based on the length of the geodesic connecting these vector distributions on a Riemannian manifold. Our tools reveal more information about these relationships than attention scores. In this study, we also show how Information-Theoretic analysis can help in troubleshooting learning problems in the Transformer layers.

## 1. Introduction

Although there is abundant literature describing the operations performed by each layer of the Transformer model, an information-theoretical understanding of how information is encoded and processed within these layers is scarce. In this study, we take a deeper look at the Transformer through the lens of Information Theory. There are several transformer-based models that are actively in use in research and in the industry. However, in this study, we used the model from the paper that introduced the Transformer architecture [1]. The information-theoretical techniques that we use in our study are general enough that they can be extended to other transformer-based models.

**Figure 1:**
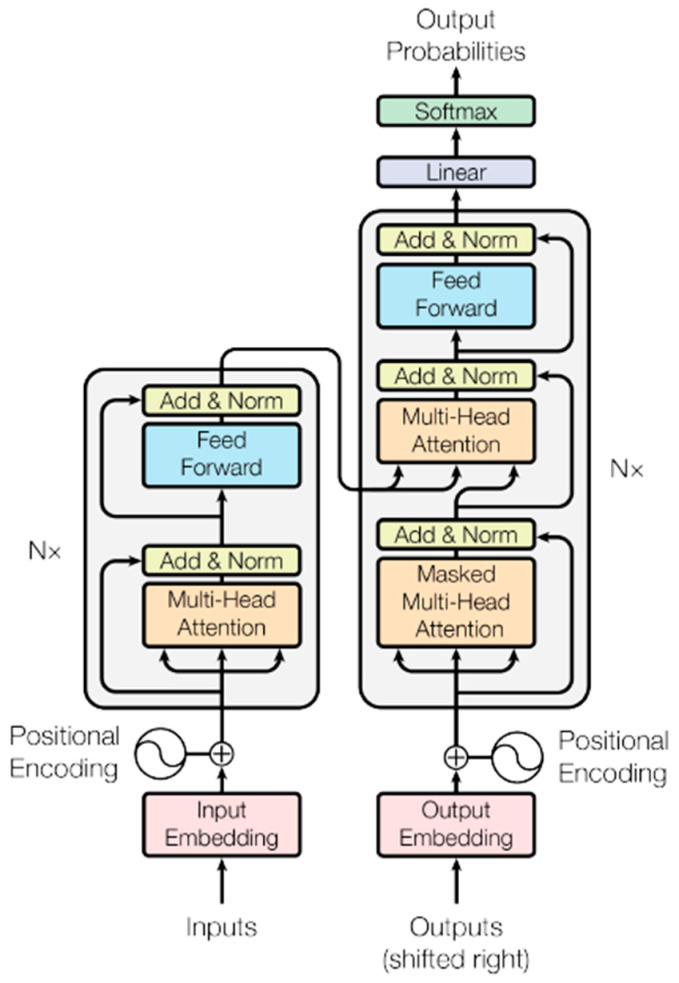
Block diagram of the Transformer model from the paper “Attention is all you need” [1].

The Transformer architecture was inspired by the ability of Recurrent Neural Networks (RNN) to translate words from English to French [2]. However, the Transformer improved on the RNN architecture by introducing the self-attention mechanism that can operate on multiple words of the input in parallel. Figure 1, taken from the introductory Transformer paper, illustrates the various blocks of the model. In our analysis, we capture the input and output vectors and matrices passing through the various layers and the various blocks within the layers of the Transformer. After each epoch of training, the data capture operation is activated while the Transformer model is switched to evaluation mode and the validation dataset is sent through it.

We study how the information in the input vectors is mapped to a high-dimensional vector space by projection matrices and analyze the characteristics of these matrices. The Transformer treats these projections as sufficient statistics of the input parameter that the model learns during the training phase. The analysis of these matrices provides interesting insight into the generative techniques used by the Transformer. This paper is divided into two main parts: Part 1 presents the Information Theoretic Analysis of high-dimensional vectors of the Transformer in Euclidean space, and Part 2 analyzes the Transformer vectors using Information Geometry on a Riemannian manifold. In the first part, we compare the Mutual Information (MI) between the word token vectors projected to a high-dimensional Euclidean space to show the complex relationship between the words that the Transformer has learnt. We visualize the relationship in an Information Plane spanned by measures like the Mutual Information and the Wasserstein distance to show the relationship between the probability distribution of the vector coordinates and the distance between these distributions. The visualization of the encoded relationship in probability space, using measures such as the Jensen-Shannon divergence and the Bhattacharya coefficient, reveals the amount of overlap between the vector’s probability density function and the distance between these density functions. These visualizations expose linear and non-linear relationships between the words of the input sentences that the Transformer model encodes in a high-dimensional vector space. Our study compares the attention scores with the MI of the input word vectors and shows that although both show similar relationships between the words in a sentence, MI can expose some weaker relationships that are not visible with the attention scores. Since our Information Theoretical tools are based on the probability distribution of the vector coordinates, they expose higher order statistics about these relationships compared to the attention scores, which are based on the vector dot-product. To quantify the amount of information passing through a specific block of the Transformer, we view the block as an information transmission channel and compute the conditional probability of the output symbol given the input symbol, and then compute the capacity of the channel. Through an example, we present how Information Theoretic tools can be used to troubleshoot issues with learning in any of the Transformer layers. The second part of this paper analyzes the high-dimensional vectors of the Transformer using Information Geometry. In this section, the vector distributions are viewed as points on a high-dimensional Riemann manifold. The Riemann manifold is a differential manifold and is endowed with a metric called the Fisher metric, which enables us to perform calculus on this manifold. We use this metric to numerically compute the geodesic, which is the shortest line connecting the two probability distribution points on the curved Riemann manifold. From the length of the geodesics, we infer the relationship between the words that the Transformer encodes in a high-dimensional vector space. The structure of the rest of the sections is the following: Section 2 describes the methods and tools used for our Mutual Information and Information Plane-based analysis, where the vector distributions are viewed in Euclidean space; Section 3 contains the results of our Mutual Information analysis on the various layers of the Transformer. Section 4 describes how to troubleshoot learning problems in the Transformer layers based on Information Theoretic tools described in this study, Section 5 analyzes the Transformer vectors using Information Geometry on a Riemannian manifold and also contains the results of this analysis, Section 6 discusses our observations, and Section 7 lists the conclusion.

## 2. Methods Used for the Information Theoretical Analysis in Euclidean Space

In this section, we explain the techniques to estimate the joint and conditional Probability Density Function (PDF) between two random vectors and compute the MI between them. These techniques form the basis of computing other metrics in our study, such as the Entropy, Kullback-Leibler divergence (KLD), the Jensen-Shannon divergence (JSD), the Wasserstein distance, and the Bhattacharya coefficient. Studies which have used MI estimators include [3,4,5,6].

We evaluated three different methods to estimate the MI between two random variables: Kraskov-Stögbauer-Grassberger (KSG) estimator, Mutual Information Neural Estimator (MINE), and the Kernel Density estimator (KDE). We compared the output of each of these estimators and confirmed that in most cases the MI values match within ±0.75 bits. In our study, we were interested in the relative MI between word token vectors, and the absolute MI numbers are not as important as the relative MI between the vectors. To prevent any systematic bias in our MI estimation, we verified the MI estimates in our study using all three methods. Although the absolute numbers differed slightly between these methods, the relative estimates across all the vectors measured remained consistent. The strengths and weaknesses of the KSG estimator and the MINE estimator have been described in [7] and [8] respectively. The KDE is evaluated in [9].

**Figure 2:**
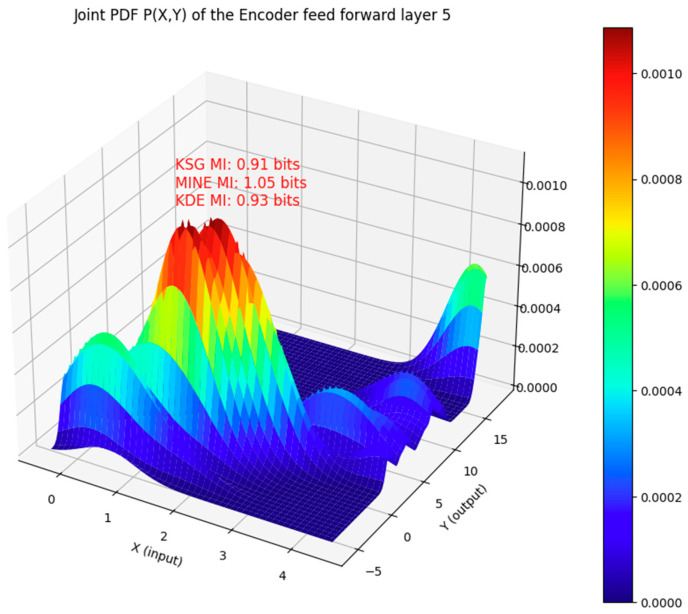
Comparison of the MI estimate with the KSG, MINE, and KDE methods for the input and output vectors that are jointly distributed as shown. The input and output vectors are from the feedforward neural network of the layer 5 encoder sub-layer of the Transformer model. In this case, the mutual information computed by all three estimators is within ±0.14 bits of each other.

Figure 2 shows the joint PDF of the input and output of the feedforward block of the encoder’s sub-layer 5 of the Transformer model. The vectors are slices along the 347th column of the input and output matrices of this block. The figure shows that the MI estimate output by each of these three methods is within ±0.14 bits.

### 2.1. Kraskov-Stögbauer-Grassberger (KSG) Estimator of the Mutual Information

The KSG estimator [10] places the smallest rectangular kernel on each sample i that contains k neighboring samples. For this study, the N samples are from a bivariate distribution of random variables X and Y. If $\varepsilon_{x}\left(i\right)\;$is the length of the rectangle’s side along the *x*-axis, $\varepsilon_{y}\left(i\right)\;$is the length of the side along the y-axis and $n_{x}\left(i\right),n_{y}\left(i\right)$ are the number of samples such that $\left\|x_{i}-x_{j}\right\|\leq \varepsilon_{x}^{\left(k\right)}\left(i\right)/2$ and $\left\|y_{i}-y_{j}\right\|\leq \varepsilon_{y}^{\left(k\right)}\left(i\right)/2$ respectively, then the KSG Mutual Information is estimated using the equation:

(1) $$\hat{I}\left(X;Y\right)=\psi \left(k\right)-1/k-\langle \psi \left(n_{x}\right)+\psi \left(n_{y}\right)\rangle +\psi \left(N\right)$$

where $\psi \left(x\right)=\Gamma^{-1}\left(x\right)\frac{d\Gamma }{dx}$ is the digamma function which satisfies the recursion $\psi \left(x+1\right)=\psi \left(x\right)+1/x$ and $\psi \left(1\right)=0.5772156\ldots$ The digamma function arises from the expected value of $\log\left(p_{i}\right)$ where $p_{i}$ is the mass of the ε—rectangle centered around the sample i. This estimator also estimates the joint and marginal entropies $H\left(X,Y\right),H\left(X\right),H\left(Y\right)$ using the same principles of k—nearest neighbor. The KSG estimator takes a free parameter k; in our study, we used the value k=8, which is relatively high. This value was chosen due to the sparsity of the sample distribution within the large vector space of the Transformer model blocks.

### 2.2. Mutual Information Neural Estimator (MINE)

The MINE [11] method uses a neural network to estimate the MI $I\left(X;Y\right)$ from the sample distribution of the X and Y random variables. It does this by using the following Donsker-Varadhan [12] variational form of the MI:

(2) $$I\left(X;Y\right)=\underset{T:Χ\times Y\to R}{\sup}E_{P_{XY}}\left[T\left(X,Y\right)\right]-\log\left(E_{P_{X}P_{Y}}\left[e^{T\left(X,Y\right)}\right]\right)$$

In the above equation, $\;T\left(X,Y\right)$ is any function of the random variables X,Y such that the above expectations are finite. The evaluation of this variational form of the MI requires knowledge of the joint distribution $P_{XY}$ and the marginal distributions $P_{X},$ $P_{Y}$ which are not available in practice. To overcome this, the MINE algorithm assumes that the input samples are IID from the joint distribution $P_{XY}$ If the number of samples are large enough, then from the Law of Large Numbers, the first expectation term in the equation can be approximated as:

(3) $$E_{P_{XY}}\left[T\left(X,Y\right)\right]\approx \frac{1}{N}\sum_{i=1}^{N}T\left(X_{i},Y_{i}\right)$$

The input samples which are drawn according to the distribution $P_{XY}$ cannot be directly used to compute the second expectation term in the Donsker-Varadhan equation since this expectation is over the distribution $P_{X}P_{Y}$. To overcome this, tuples $\left(X,\tilde{Y}\right)$ are synthetically constructed by randomly shuffling the Y samples from the input sample tuples $\left(X,Y\right)$. This makes X and $\tilde{Y}$ statistically independent and IID distributed as $P_{X}P_{Y}$. The Law of Large Numbers is then applied to the tuples $\left(X,\tilde{Y}\right)$ to approximate the second expectation term in (2):

(4) $$\log\left(E_{P_{X}P_{Y}}\left[e^{T\left(X,Y\right)}\right]\right)\approx \log\left[\frac{1}{N}\sum_{i=1}^{N}e^{T\left(X_{i},{\tilde{Y}}_{i}\right)}\right]$$

Substituting (3) and (4) in (2) the MI estimator can be expressed as:

(5) $$\hat{I}\left(X;Y\right)=\underset{T:Χ\times Y\to R}{\sup}\frac{1}{N}\sum_{i=1}^{N}T\left(X_{i},Y_{i}\right)-\log\left[\frac{1}{N}\sum_{i=1}^{N}e^{T\left(X_{i},{\tilde{Y}}_{i}\right)}\right]$$

To maximize this equation over all functions $T\left(X,Y\right)$, the MINE method employs a neural network $T_{\theta }\left(X,Y\right)$ whose cost function is defined by (5).

### 2.3. Kernel Density Estimator (KDE) of the Mutual Information

The KDE method is a non-parametric estimator of the PDF. It places a kernel over each sample of the distribution to generate a set of weighted samples and creates a smooth estimate of the PDF by taking an average of the weighted samples. For the bivariate distribution in our study, the KDE estimate of the PDF is expressed as:

(6) $$\hat{p}\left(z\right)=\frac{1}{N}\sum_{i-1}^{N}K\left(z-z_{i}\right)$$

where $z_{i}=\left(x,y\right)$ is the tuple of input samples of random variables X and Y. K in this equation is the kernel function. We used a Gaussian kernel since it has infinite support. The sample size in our model was large but sparse over the large vector space of the various Transformer blocks, and as a result, we did not see any significant performance improvement with other kernels such as Epanechnikov, exponential, tophat, and cosine. The expression for the Gaussian kernel is:

(7) $$K\left(z\right)=\frac{1}{2\pi \sqrt{\left|H\right|}}e^{-\frac{1}{2}z^{T}H^{-1}z}$$

where H is a 2×2 matrix called the kernel bandwidth. The kernel bandwidth controls the amount of smoothing applied to the weighted samples in the 2-D distribution space. The proper choice of this parameter is critical to the accuracy of the PDF estimator. To select the optimal value for the bandwidth parameter we performed a k—fold cross-validation with k=5. The cross-validation was performed over a range of bandwidth values, and the best bandwidth was selected based on the log-likelihood of the test data for that bandwidth. If the cross-validation algorithm selected a bandwidth value that was any of the values at the limits of the range, the range was automatically increased, and the cross-validation was re-run.

The joint PDF estimate $\hat{p}\left(x,y\right)$ obtained from the KDE was used to compute the following marginal PDFs using numerical integration (i.e., Simpson’s rule):

(8) $$\begin{array}{l} \hat{p}\left(x\right)=\int_{y}\hat{p}\left(x,y\right)dy\\ \hat{p}\left(y\right)=\int_{x}\hat{p}\left(x,y\right)dx \end{array}$$

From these PDF estimates, the following joint and marginal entropy estimates were calculated using numerical integration:

(9) $$\begin{array}{l} \hat{H}\left(X,Y\right)=\int_{x}\int_{y}\hat{p}\left(x,y\right)\log_{2}\hat{p}\left(x,y\right)dxdy\\ \hat{H}\left(X\right)=\int_{y}\hat{p}\left(x\right)\log_{2}\hat{p}\left(x\right)dx\\ \hat{H}\left(Y\right)=\int_{x}\hat{p}\left(y\right)\log_{2}\hat{p}\left(y\right)dy \end{array}$$

With the entropy estimates obtained from (9) we used the following equation to estimate the mutual information between the random variables X and Y:

(10) $$\hat{I}\left(X;Y\right)=\hat{H}\left(X\right)+\hat{H}\left(Y\right)-\hat{H}\left(X,Y\right)$$

The Kullback-Leibler divergence between PDF estimates $\hat{p}\left(x\right)$ and $\hat{q}\left(x\right)$ was computed, by evaluating the following integration numerically:

(11) $$D_{KL}\left(\hat{p}\left(x\right)\left\|\hat{q}\left(x\right)\right.\right)=\int_{x}\hat{p}\left(x\right)\log_{2}\left[\frac{\hat{p}\left(x\right)}{\hat{q}\left(x\right)}\right]dx$$

In our analysis, we used the following expression for the Jensen-Shannon divergence (JSD):

(12) $$D_{JS}\left(\hat{p}\left(x\right)\left\|\hat{q}\left(x\right)\right.\right)=\frac{1}{2}D_{KL}\left(\hat{p}\left(x\right)\left\|\frac{\hat{p}\left(x\right)+\hat{q}\left(x\right)}{2}\right.\right)+\frac{1}{2}D_{KL}\left(\hat{q}\left(x\right)\left\|\frac{\hat{p}\left(x\right)+\hat{q}\left(x\right)}{2}\right.\right)$$

If the base of the logarithm used to calculate the Jensen-Shannon divergence is 2, then:

(13) $$0\leq D_{JS}\left(\hat{p}\left(x\right)\left\|\hat{q}\left(x\right)\right.\right)\leq 1$$

The JSD is a statistical measure of how much the probability distributions differ. The JSD is closer to 0 when the distributions are similar.

We viewed the blocks of the Transformer as Shannon information channels. For example, the feedforward block of each attention layer takes a matrix as input and outputs a matrix of the same size. The amount of information that passes through this channel is bounded by the channel’s information-carrying capacity. From Shannon’s channel coding theorem, the capacity of the channel with random variable X as input and Y as output, is given by:

$$C=\underset{P_{x}}{\sup}I\left(X;Y\right)$$

To compute the information carrying capacity of this channel, we first estimated the following conditional probability using the PDF estimates in (8):

(14) $$\hat{p}\left(y\left|x\right.\right)=\frac{\hat{p}\left(x,y\right)}{\hat{p}\left(x\right)}$$

Then we used the Blahut-Arimoto algorithm [13] to compute the information channel capacity. This is an iterative algorithm that takes as input the conditional probability $\hat{p}\left(y\left|x\right.\right)$ and iteratively converges to the capacity of the channel.

### 2.4. Probability Distribution Distance Metrics for the Information Plane

To view the high-dimensional vectors on an Information Plane, we use the following distance metrics: Bhattacharya coefficient and the Wasserstein distance.

If $p\left(x\right)$ and $q\left(x\right)$ are two probability distributions on the same domain X, then the Bhattacharya coefficient [14,15] is defined as:

(15) $$BC\left(p,q\right)=\int_{x\in X}\sqrt{p\left(x\right)q\left(x\right)}dx$$

For discrete probability distributions, the Bhattacharya coefficient is expressed as:

(16) $$BC\left(p,q\right)=\sum_{x\in X}\sqrt{p\left(x\right)q\left(x\right)}$$

The Bhattacharya coefficient quantifies how much the probability distributions $p\left(x\right)$ and $q\left(x\right)$ overlap. It is therefore a measure of the statistical similarity of the distributions. The Bhattacharya coefficient can only take values in the interval [0,1].

Let $\left(M,d\right)$ be a metric space that is a separable and complete topological space, and let P and Q be two probability measures on M. Let $\pi \left(P,Q\right)$ be a set of all couplings of P and Q. A coupling γ is a joint probability measure M×M whose marginals are given by P and Q. Then the Wasserstein *p*-distance [16] is defined as:

(17) $$W_{p}\left(P,Q\right)=\underset{\gamma \in \pi \left(P,Q\right)}{\inf}{\left[E_{\left(x,y\right)∼\gamma }d{\left(x,y\right)}^{p}\right]}^{1/p}$$

We use p=1 in our study, which makes the Wasserstein distance the same as the Earth Mover distance, and the minimization problem in the above equation can be viewed as an optimal transport problem. To understand the optimal transport problem, consider a 2-dimensional space consisting of N grid points. Each grid point position can be denoted as ${\left\{x_{i}\right\}}_{i=1,\ldots N}$. Let some of the grid points contain dirt (or earth) whose mass is given by the probability $p_{i}$, and some of the grid points contain holes whose capacity to contain dirt is given by the probability $q_{i}$. The dirt must be moved from the grid points containing dirt to the grid points with holes so that there are no grid points with piles of dirt left. If P is the total mass (or total probability) of the dirt and Q is the total capacity (or total probability) of the holes, then we can write the following expression for P and Q:

(18) $$P=\sum_{i=1}^{N}p_{i}\delta_{x_{i}}\;\mathrm{and}\;Q=\sum_{i=1}^{N}q_{i}\delta_{x_{i}}$$

In the above expression $\delta_{x_{i}}$ is the Dirac delta function placed at location $x_{i}$ in the 2-dimensional grid (i.e., $x\in {\mathbb{R}}^{2}$). Let the cost of moving one unit of dirt from bin i to bin j or vice-versa be $C_{ij}$. This cost could be a distance metric like $C_{ij}={\left\|x_{i}-x_{j}\right\|}^{2}$. The transport plan $\pi_{ij}\left(P,Q\right)$ has the following constraint when dirt is moved from location $x_{i}$ to one or more locations $x_{j}$:

(19) $$\sum_{j}\pi_{ij}=p_{i}$$

Also, when dirt is moved to a location $x_{j}$ from any location $x_{i}$ the transport plan has the following additional constraint:

(20) $$\sum_{i}\pi_{ij}=q_{j}$$

The Wasserstein distance can now be formulated as the following minimization problem:

(21) $$W\left(P,Q\right)=\underset{\pi_{ij}}{\min}\left\{\sum_{i=1}^{N}\sum_{j=1}^{N}\pi_{ij}C_{ij}:\;\pi_{ij}\geq 0,\sum_{j}\pi_{ij}=p_{i},\sum_{i}\pi_{ij}=q_{j}\right\}$$

Intuitively, the Wasserstein distance is the minimum amount of work required to move one probability distribution to another to make them the same. Therefore, the Wasserstein distance is a measure of dissimilarity between two probability distributions.

## 3. Analysis of the Transformer Model

The model in the paper [1] that introduced the Transformer architecture comprises an encoder and decoder as shown in Figure 1. We implemented this exact model with the parameters listed in the paper. With our implementation, we could add hooks in every layer of the model to capture the inputs and outputs. Our implementation of the Transformer model was trained on the English-French dataset [17], which is composed of 127,085 input paragraphs. Each paragraph contains one or more sentences in English along with the French translation of these sentences. The vocabulary that was generated from the dataset comprised 30,000 words. Just like the introductory Transformer paper, we used an Adam optimizer with a learning rate of $10^{-4}$ and $\varepsilon =10^{-9}$. For regularization, we used a dropout rate of 0.1. We used 90% of the dataset for training and the rest for validation. The Transformer was trained over 20 epochs with a batch size of 8. After the training, the Transformer was able to translate English sentences to French from the validation dataset, with a BLEU score of about 0.45.

The encoder has 6 sub-layers, each of which has a multi-head attention block followed by a fully connected feed-forward neural network block. The outputs of these blocks are normalized and connected to their inputs via residual connections. The input sentences to the Transformer are tokenized, which is a procedure where each word in the sentence is assigned a unique integer from the vocabulary of words. These tokens are passed through an input embedding layer, which maps each token to a vector of length $d_{\mathrm{model}}$. The hyperparameter $d_{\mathrm{model}}$ is equal to 512 in the paper, and one or more of the dimensions of all the vectors and matrices that we analyzed in this study are controlled by this hyperparameter. During the probing, the model was set to ‘evaluation mode’ so that the model weights did not change during this operation. Therefore, our probing data captured the behavior of the Transformer after each epoch of training was completed. We used a batch length of 1 during the probing.

In the following subsections, we describe each layer of the encoder and decoder in detail and include our information-theoretical analysis of the mechanics of the various blocks in each of these layers.

### 3.1. Input Embedding Layer

**Figure 3:**
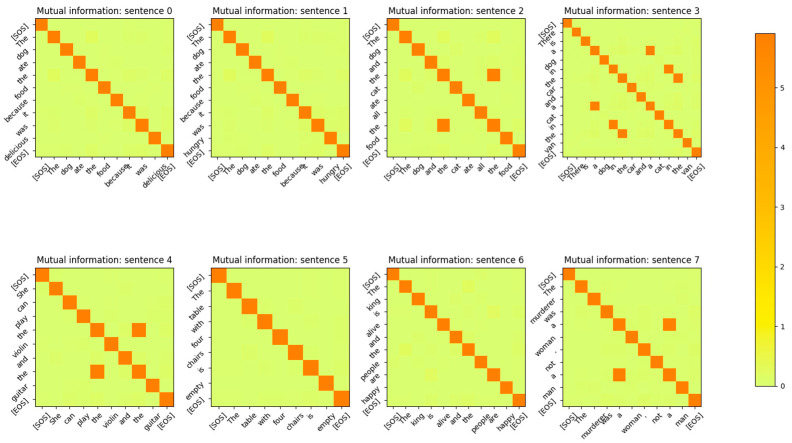
Mutual Information between the Input Embedding output vectors for $d_{\mathrm{model}}=512$ at the end of the last epoch of training. The input sentences are from the validation set. The same tokens, irrespective of the position in the sentence, have high MI. Embedding vectors for different tokens are mutually independent. The diagonal represents the entropy of the token. Some token vectors that correspond to the same word but at a different position in the sentence have a high MI with the token at a different position. This shows that no positional information is encoded in the word embedding layer.

The Input Embedding layer is a neural network that learns its weights during the training phase of the Transformer. The Embedding layer maps the input token integers to vectors of length $d_{\mathrm{model}}$. The number of words/tokens in the sentence is denoted by sequence_length. The input to the Embedding layer during probing is a vector of dimension (1 × sequence_length) and is composed of integers corresponding to the token ID assigned to each word of the sentence. The output of the input embedding layer is a matrix of dimension (sequence_length $\times \;d_{\mathrm{model}}$) during the probing phase. The MI between the vectors output by the input embedding layer for each token of the input sentences is shown in Figure 3. The sentences in this figure were part of the validation set of the Transformer. From the figure, it is apparent that the input embedding layer maps all the input tokens of a sentence to mutually independent vectors. In other words, the MI between any two embedding vectors of a sentence is close to 0 unless the vectors correspond to the same input token.

**Figure 4:**
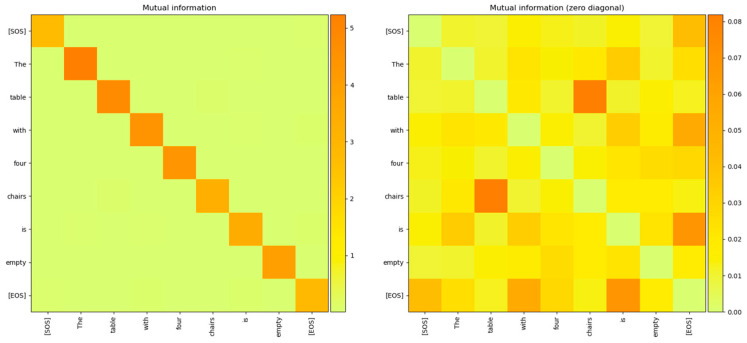
The mutual information between the word embedding vectors plotted with and without the diagonal zeroed out to expose the low values of the Mutual Information between different token vectors. The picture on the right is with the diagonals zeroed out and it clearly shows that the mutual information of the token vector distribution with other vectors is very low, making the distributions independent of each other.

The MI between the word embedding vectors for one of the sentences is shown in Figure 4, with the diagonal entries (corresponding to the entropy of the vector distribution) zeroed out so that the matrix entries with low MI values are visible. From this figure, it is evident that the MI between different word embedding vector distributions is very low and the distributions are independent of each other.

**Figure 5:**
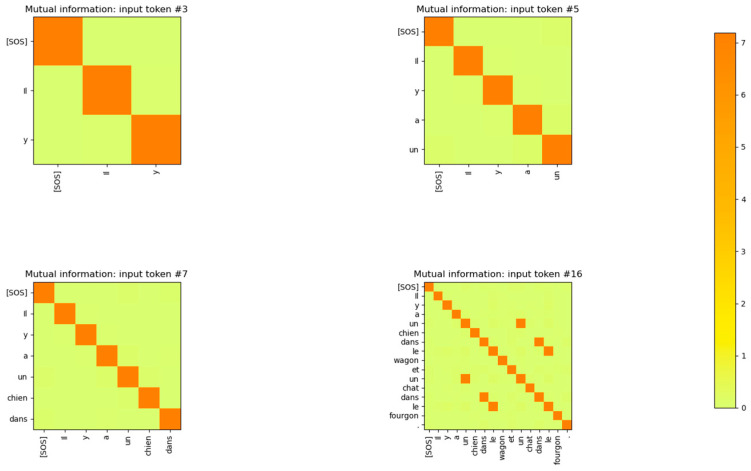
MI between the rows of the decoder’s word embedding layer output matrix for each token input to this layer. The decoder’s output is concatenated with the previous output and fed as input to the word embedding layer. It is evident that the output vectors of different tokens are mutually independent from each other.

The encoding by the word embedding layer has no positional information, as the same words at different positions of the sentence have high MI. The embedding layer is able to learn weights that generate mutually independent vectors for each word token because the output vectors are mapped to a high-dimensional vector space ($d_{\mathrm{model}}=512$). In Section 4, we show that if the model dimensions are reduced, the Embedding layer neural network is no longer able to generate word token vector distributions that are mutually independent. The decoder’s embedding layer exhibits the same behavior as shown in Figure 5.

### 3.2. Positional Encoding Layer

The Positional Encoding layer adds information pertaining to the position of the token in the input sentence. The position matrix values are deterministic (i.e., not learned during training) and are defined by the following equations:

(22) $$\begin{array}{l} \mathrm{PE}\left[n,2i\right]=\sin\left(\omega_{i}n\right)\;\mathrm{for}\;i=0,2,4,\ldots ,d_{\mathrm{model}}\\ \mathrm{PE}\left[n,2i+1\right]=\cos\left(\omega_{i}n\right)\;\mathrm{for}\;i=1,3,5,\ldots ,\left(d_{\mathrm{model}}-1\right) \end{array}$$

where n is the position of the token in the sentence and $d_{\mathrm{model}}$ is an even number. The angular frequency term in the above equation is defined as:

(23) $$\omega_{i}=\frac{1}{{10,000}^{2i/d_{\mathrm{model}}}}$$

The angular frequency can also be expressed as:

(24) $$\omega_{i}=\frac{2\pi }{\lambda_{i}}$$

where $\lambda_{i}$ is the wavelength of the sine or cosine. Equating (23) and (24) we have:

(25) $$\begin{array}{l} \omega_{i}=\frac{1}{{10,000}^{2i/d_{\mathrm{model}}}}=\frac{2\pi }{\lambda_{i}}\\ ∴\lambda_{i}=2\pi \left({10,000}^{2i/d_{\mathrm{model}}}\right) \end{array}$$

Therefore, the wavelengths of the sinusoids used in the positional encoding matrix form a geometric progression from 2π to $10,000\left(2\pi \right)$.

A word token at position n in the sentence is related to another word at position n+Δ by a simple rotation matrix M expressed as:

(26) $$M=\left(\begin{array}{cc} \cos\left(\omega_{k}\Delta \right) & \sin\left(\omega_{k}\Delta \right)\\ -\sin\left(\omega_{k}\Delta \right) & \cos\left(\omega_{k}\Delta \right) \end{array}\right)$$

**Figure 6:**
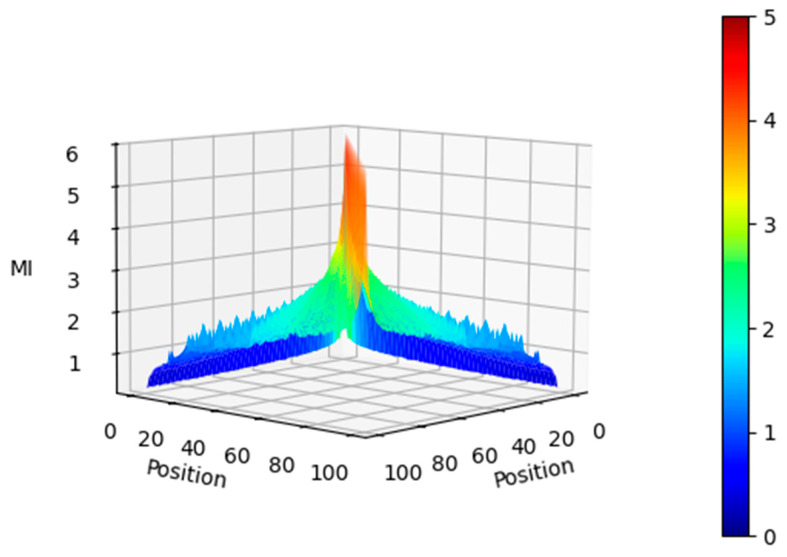
Mutual Information between the Positional Encoding vectors for seq length = 100 and $d_{\mathrm{model}}=512$ (side view). The MI gradually tapers off with the position delta between tokens.

**Figure 7:**
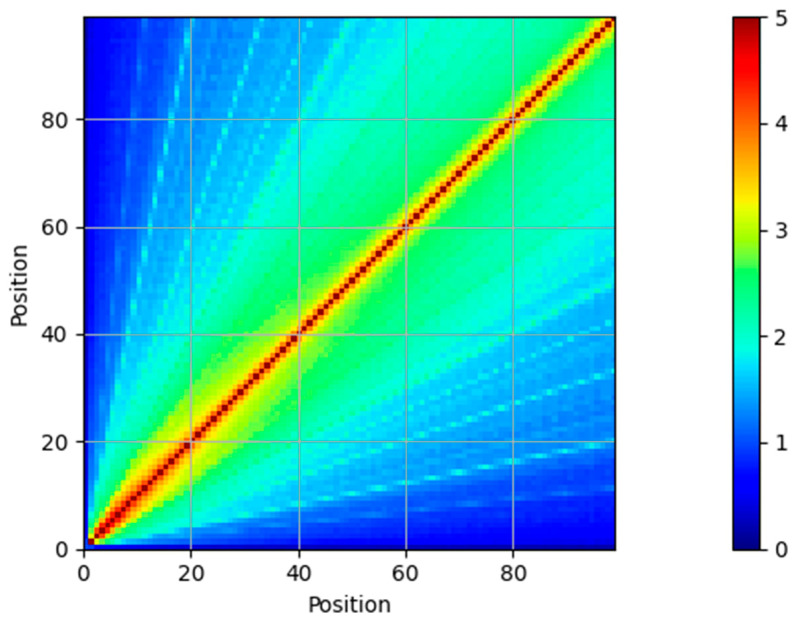
Mutual Information between the Positional Encoding vectors for seq length = 100,$d_{\mathrm{model}}=512$ (top view). Tokens that are 20 positions away have an MI of about 3 bits.

The projection matrices are square matricesThis relationship can be seen in the MI between the elements of the positional encoding vector in Figure 6 and Figure 7.

As shown in the figures, the MI is high (5 bits) between the vectors at the same position of the positional encoding matrix, and it gradually tapers off. Even at a distance of 20 positions, the MI is about 3 bits. This implies that the relationship between tokens in a sentence that are 20 positions away can be encoded in the Positional encoding vectors. An interesting observation from Figure 7 is that tokens at a distance of 3 and 5 times the current token position have a relatively high MI with each other. This is visible in the cyan lines of slopes 3 and 5 in the bottom plots. This is due to the geometric progression of the wavelength of the sinusoids, and the MI of the positional encoding vectors clearly exposes this.

**Figure 8:**
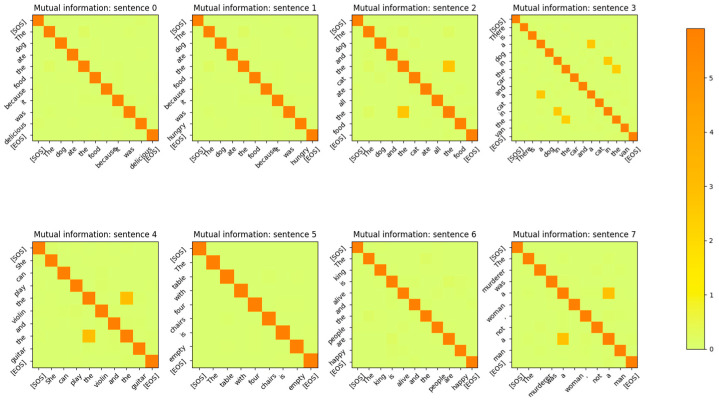
Mutual Information between the vectors of the Positional Encoding layer output for the sentences in the validation dataset. Comparing these plots to Figure 3 shows that the MI of the same tokens at different positions has reduced from 5 bits to 2 bits due to the addition of positional information.

The MI between the output vectors of the Positional Encoding layer is shown in Figure 8. Comparing this figure with the MI between the output vectors of Figure 3, it is evident that the addition of the positional information has reduced the MI between vectors of the same token, which are at a different position in the sentence. The MI for these tokens is still non-zero, but it is reduced to 2 bits from 5 bits after the addition of the Positional Encoding vectors. This also shows that the model has a strong desire to retain information related to the same tokens at different positions in the sentence, even though this information is reduced by the addition of the Positional Encoding vectors. The output of the embedding layer is added to the output of the positional encoding layer, resulting in a vector that contains the identity and positional information of the token in the sentence. This vector is then sent to the encoder layer for further processing.

### 3.3. Encoder and Decoder Layer

The input to the encoder and decoder is a matrix of dimension (sequence_length $\times \;d_{\mathrm{model}}$) during the probing phase. Figure 1 shows the blocks of the Encoder and Decoder. The Multi-head attention, the Feed Forward network and the Add and Norm blocks shown in the figure form just one sub-layer of the Encoder. This sub-layer is replicated 6 times in the Transformer paper and stacked on top of each other. The matrix that is input to the Encoder and Decoder is replicated as query (Q), key (K) and value (V) matrices at the input of the mutlihead attention block of each of the Encoder and Decoder sub-layers. Each of the Q,K,V matrices are of dimension (sequence_length $\times \;d_{\mathrm{model}}$). At the input of the encoder, each of them are post-multiplied by projection matrices $W_{Q},W_{K}$ and $W_{V}$ as expressed below:

(27) $$\begin{array}{l} Q\times W_{Q}=Q^{\prime }\\ K\times W_{K}=K^{\prime }\\ V\times W_{V}=V^{\prime } \end{array}$$

**Figure 9:**
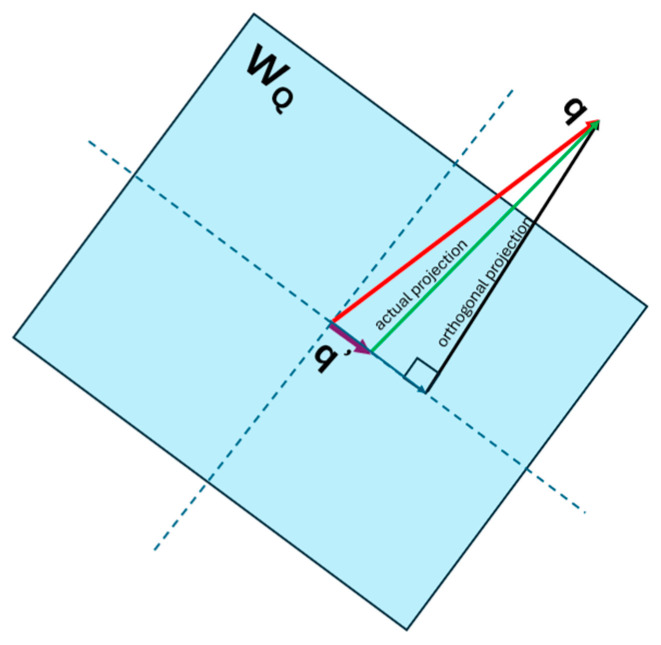
Projection of the input vector q to the column space of $W_{Q}$ is not orthogonal.

The projection matrices are square matrices of dimension $\left(d_{\mathrm{model}}\times d_{\mathrm{model}}\right)$ and the dimension of the projections $Q^{\prime },K^{\prime },V^{\prime }$ is (sequence_length $\times \;d_{\mathrm{model}}$). Even though the Transformer paper calls these matrices projection matrices, we found that these matrices are not idempotent. Therefore, each row vector $q^{\prime },k^{\prime },v^{\prime }$ of the matrix $Q^{\prime },K^{\prime },V^{\prime }$ does not lie fully in the vector space spanned by the columns of $W_{Q},W_{K},W_{V}$. We verified that the matrices $W_{Q},W_{K},W_{V}$ achieve full rank during training. Therefore, either the quality of the data or the complexity of the design is not sufficient to make these matrices idempotent. We expect the performance of the Transformer to improve with idempotent projection matrices as there would be less collision between input token vectors when they are mapped to the high-dimension space by these projection matrices. Another thing to note about these projection matrices is that they are not symmetric and consequently their eigenvalues are not real and have values other than 0 and 1. This implies that the projection of the input vectors q,k,v to the space spanned by the columns of $W_{Q},W_{K},W_{V}$ are not orthogonal. As a result, the projected vectors $q^{\prime },k^{\prime },v^{\prime }$ move further away from the input vectors q,k,v when they are projected to the column space of $W_{Q},W_{K},W_{V}$. This is illustrated for the input vector q in Figure 9.

**Figure 10:**
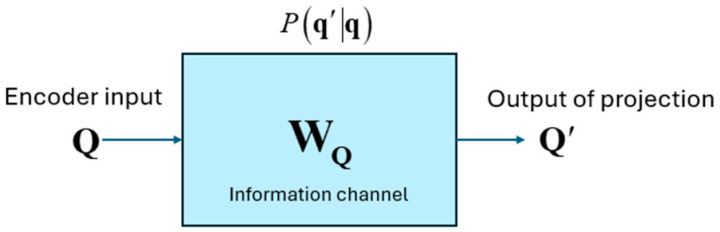
Projection matrix $W_{Q}$ as an information channel.

This implies that the projection not only maps the input vectors to a different vector space, but it also changes the norm of the input vectors as part of the generalization process during learning. In order to quantify the amount of information in the input vectors that passes through the projection matrices we viewed the projection matrices $W_{Q},W_{K},W_{V}$ as information channels and estimated the conditional probability $P\left(q^{\prime }\left|q\right.\right)$ between the input q,k,v vectors and the output $q^{\prime },k^{\prime },v^{\prime }$ for input and output symbols quantized to 100 different values (i.e., 10,000 input and output combinations of discrete symbols). The information channel for $W_{Q}$ is illustrated in Figure 10. We computed the channel capacity using the Blahut-Arimoto algorithm to determine the number of bits of information that pass through from the input of this projection block to its output.

**Figure 11:**
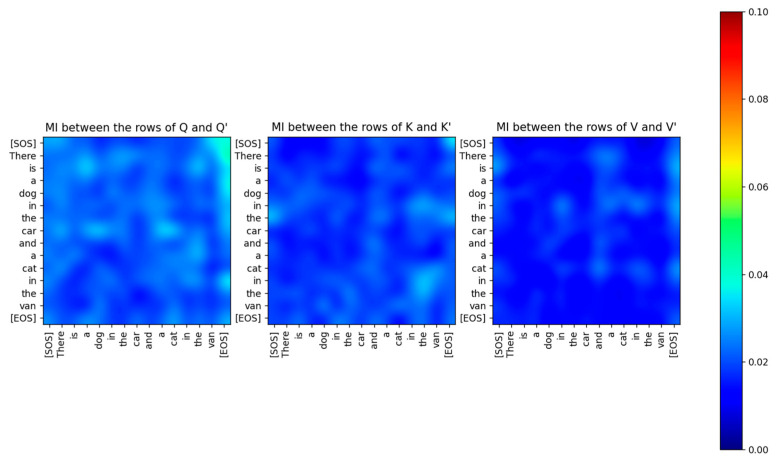
The Mutual Information (MI) between the rows of Q,K,V and $Q^{\prime },K^{\prime },V^{\prime }$ for attention layer 2 for a specific sentence. The MI is very low even between the same tokens which shows that the vectors q,k,v and $q^{\prime },k^{\prime },v^{\prime }$ are mutually independent after the projection. All the attention layers show the same behavior.

As we expected, the capacity of this information channel was a very small number, only about 0.6 bits. As shown in Figure 11, the MI between the rows of Q,K,V and the rows of $Q^{\prime },K^{\prime },V^{\prime }$ is very small (<0.04 bits) even for the same tokens.

**Figure 12:**
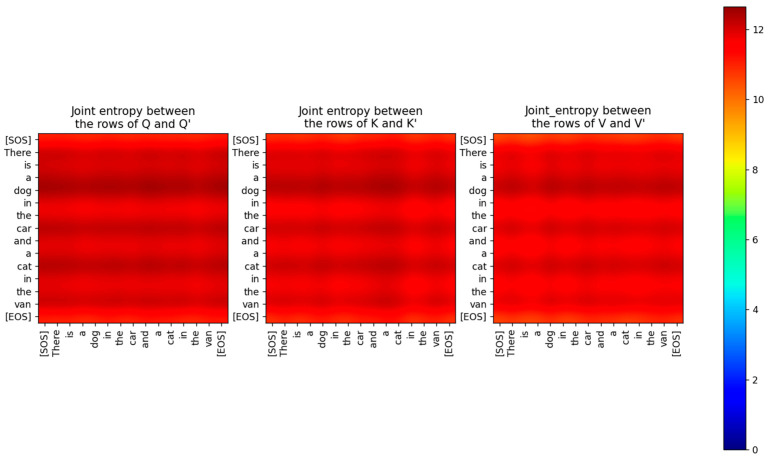
Joint Entropy between Q, K, V and Q’, K’, V’ respectively.

**Figure 13:**
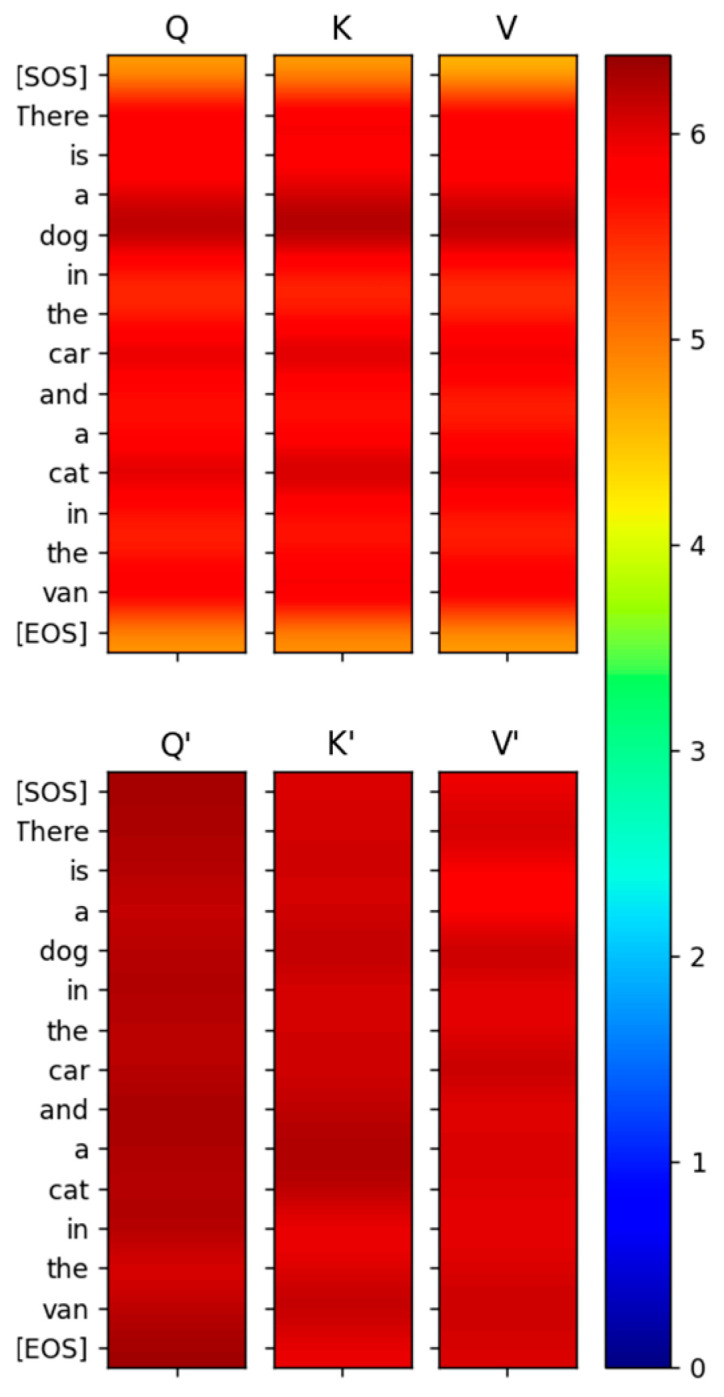
Entropy of the rows of Q, K, V and Q’, K’, V’ is high indicating high information content.

Just because the capacity of the projection matrix information channel is only 0.6 bits, it does not mean that information does not flow through this channel. The low values of the MI between the input and output vectors imply that the distributions of the input and output vectors are completely different. The joint entropy between the rows of Q,K,V and the rows of $Q^{\prime },K^{\prime },V^{\prime }$ has a high value (about 12 bits), as shown in Figure 12. The entropy of the rows of Q,K,V and the rows of $Q^{\prime },K^{\prime },V^{\prime }$ shown in Figure 13 is also high (about 6 bits).

From these MI and entropy results, it is evident that even though the channel $W_{Q}$ does not pass any part of the input to the output unaltered, there is a substantial amount of information (greater than 6 bits) at the output of the projection. The other channels $W_{K}$ and $W_{V}$ exhibit similar behavior. This is the hallmark of a generative model compared to a discriminative model like a Convolutional Neural Network (CNN). A CNN filters the input and passes the relevant part of the input to the output, such that there is significantly high MI between the input and output pertaining to the relevant information required for classification [18]. The non-zero MI between the input and output of a CNN layer reflects how much of the information from the input is still retained in the output. A generative model, on the other hand, projects the input to a high-dimensional subspace such that the MI between the input and output is almost zero. A generative model learns the parameters (or statistics) of the input by projecting it to high vector dimensions so that it can generate new data based on these statistics.

Let θ be some parameter of the input X that the Transformer is trying to learn and let the joint distribution of the input and this parameter be $P\left(X,\theta \right)$. Consider the function $T_{Q}\left(X\right),T_{K}\left(X\right),T_{W}\left(X\right)$ which project the input X to the column space of the projection matrix $W_{Q},W_{K},W_{V}$. These functions can be expressed as:

(28) $$\begin{array}{l} T_{Q}\left(X\right)=X\times W_{Q}=Q^{\prime }\\ T_{K}\left(X\right)=X\times W_{K}=K^{\prime }\\ T_{V}\left(X\right)=X\times W_{V}=V^{\prime } \end{array}$$

Since the Transformer infers this parameter θ only through the transformations $T_{Q}\left(X\right),T_{K}\left(X\right),T_{W}\left(X\right)$, it operates under the assumption that these transformation functions are a joint sufficient statistic of this parameter. In other words, if $P\left(\theta \left|X\right.\right)$ is the conditional probability of the parameter θ given the input X, then the Transformer assumes that:

(29) $$P\left(\theta \left|X\right.\right)=P\left(\theta \left|T_{Q}\left(X\right),\right.T_{K}\left(X\right),T_{V}\left(X\right)\right)\;\mathrm{for}\;\mathrm{all}\;\mathrm{priors}\;P\left(\theta \right)$$

If the vectors denoted bThe notion that projections can be viewed as a sufficient statistic for a random vector X is also supported by the moment generating function (MGF). For a fixed column vector t, the moment generating function of a random vector X (if it exists), is defined as:

(30) $$m_{X}:{\mathbb{R}}^{n}\to \mathbb{R},\;m_{X}\left(t\right)=E\left[\exp\left(t^{T}X\right)\right]$$

If the vectors denoted by t are the different columns of the projection matrices $W_{Q},W_{K,}W_{V}$ then the above expression implies that the MGF of the random vector X can be derived from the projections of this vector onto the columns of $W_{Q},W_{K,}W_{V}$. Since the MGF contains all the moments of the random vector, it characterizes the distribution of the random vector and is related to the sufficient statistic of the model parameter.

**Figure 14:**
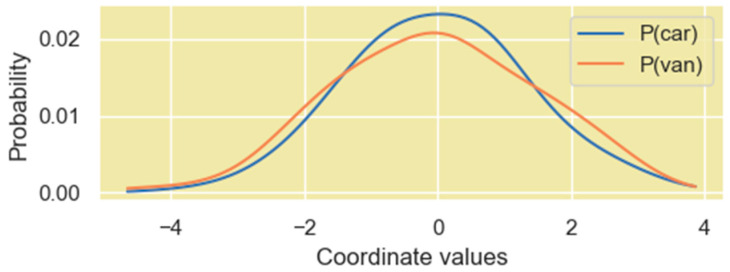
PDF of the vector coordinate values when the coordinates are viewed as numerical outcomes of a single random variable. The vectors in the plot correspond to the words “car” and “van” in the Q’ matrix.

Since our information-theoretic analysis is based on the probability distribution of the high-dimensional vectors within the Transformer layers, we view the coordinates of these vectors as samples of a distribution. One way to view the distribution of the coordinates is to simply consider them to be a set of numerical outcomes of a single random variable X. This random variable thus represents a distribution of the coordinate values of the vector, but it does not contain any mapping information of the coordinate value to the vector dimension. PDFs generated from such a distribution are shown in Figure 14 for two rows of the $Q^{\prime }$ matrix of the encoder.

The other method to view the distribution of the coordinates of these vectors is borrowed from a concept in statistical mechanics where a system consists of several microstates. The probability that the system is in a certain state is dependent on the energy of each microstate and is given by the following Boltzmann distribution expression:

(31) $$p\left(state_{i}\right)=\frac{\exp\left(-\frac{E_{i}}{kT}\right)}{\sum_{j=1}^{M}\exp\left(-\frac{E_{j}}{kT}\right)}=\frac{\exp\left(\beta E_{i}\right)}{\sum_{j=1}^{M}\exp\left(\beta E_{j}\right)}$$

**Figure 15:**
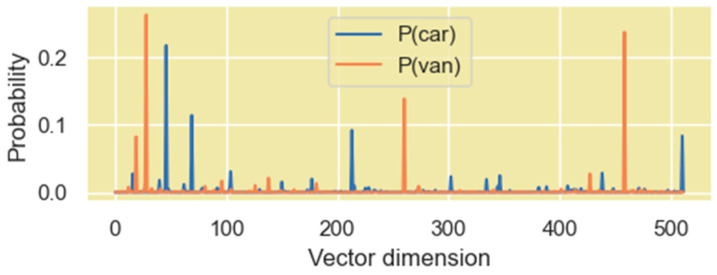
PDF of the vector coordinate values when the coordinates are viewed as energy levels of the individual microstates. The vectors correspond to the word “car” and “van” in the Q’ matrix.

In the above expression, $E_{i}$ is the energy of the microstate i and T is the temperature. This expression is like the softmax operation that machine learning models, including the Transformer model, apply to logits to generate a probability distribution. To retain the mapping information of the vector coordinates and the vector dimension, we consider each dimension of the vector as a microstate and the coordinates as the energy assigned to the specific microstate. We then generate probability values for each of these microstates (i.e., each dimension) based on the energy level used (31). In our study, we use β=2.5 to smoothen the probability distribution. We found this value of β amplifies the magnitude level of dimensions with high coordinate values just enough to make it stand out in the distribution. The PDF using this method is shown in Figure 15.

We found that both perspectives of the vector coordinate distribution are useful, and they both provide different insights on the encoding of relationships between word vectors in high-dimensional space.

**Figure 16:**
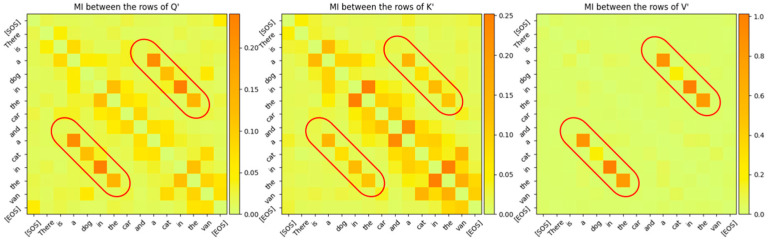
Mutual Information between the rows of Q’, K’, V’ for Attention Layer 0. The relationship between the word sequence “cat in the van” and “dog in the car” is clearly visible even though these vectors are projected to high-dimensional vector space. The diagonal entries, which are entropies of the token vectors, have been intentionally zeroed out so that the other MI values are visible.

Since $W_{Q},W_{K,}W_{V}$ are the projection matrices of the joint sufficient statistics of the input parameter, we analyzed the output of the projection to determine if the relations between words of the sentences mapped to high-dimensional space can be observed. The projected vectors corresponding to the words in the sentence that are related to each other are expected to lie closer to each other in the high-dimensional space. Although it is normally hard to observe these relationships visually due to the high-dimensional nature of the vector space, MI between the projected vectors (i.e., between rows of $Q^{\prime },K^{\prime },V^{\prime }$) clearly exposes these relationships. As shown in Figure 16, the relationship between the words in the sentence that the Transformer’s attention layer 0 is focused on is visible even when these words are projected to high-dimensional space and even before the attention scores are computed.

**Figure 17:**
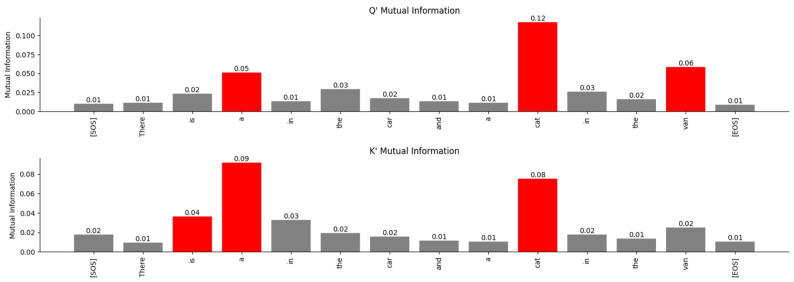
Attention layer 0 relationships between the word “dog” and the other words in the Q’ and K’ matrices in high-dimensional space exposed by MI. The Transformer associates the word “dog” with the words “a”, “cat”, “van” in the Q’ matrix and the words “is”, “a”, “cat” in the K’ matrix.

From this figure, it is evident that the Transformer’s attention is focused on a sequence of words across which it has determined a certain relationship. The bar plots in Figure 17 are based on the MI matrix plots in Figure 16 and they show the relationship between specific words and the other words in the sentence, which are all projected in high-dimensional vector space.

**Figure 18:**
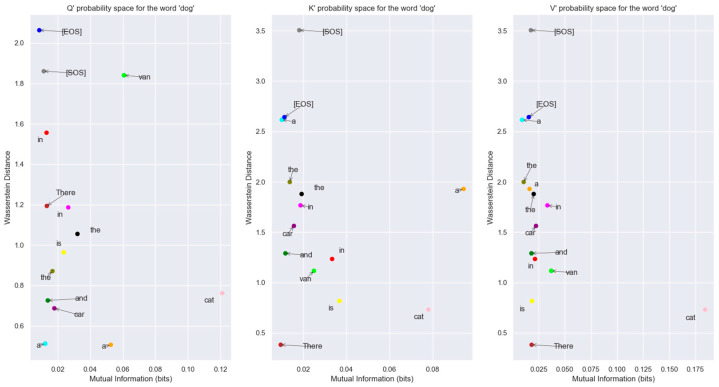
Word vectors for Q’, K’, V’ prime in The Mutual Information vs. Wasserstein distance plane. Each of the words in this plane is compared to the word vector for “dog”. Points that are closer to the bottom right part of each plot have their distributions closer to the word dog. The word “cat” is closest to the word “dog” in probability space. This view allows us to understand the relationship between words in probability space.

To visualize the relationship between the words in an Information-Theoretic plane, we plotted the vectors in the Mutual Information vs. Wasserstein distance plane in Figure 18. The relationship between the encoded words is visible in this figure.

**Figure 19:**
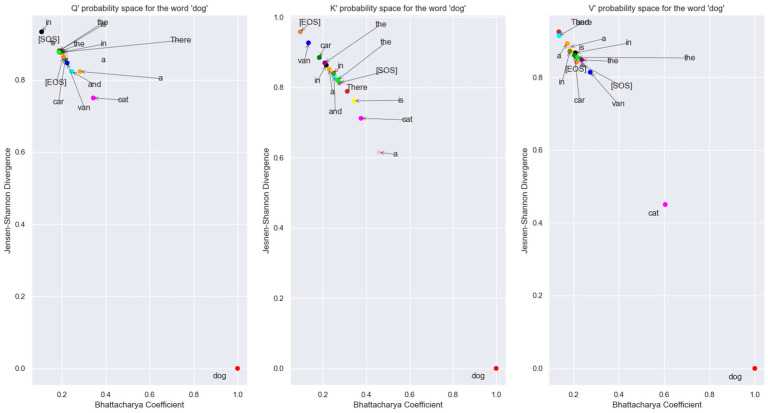
Plot of the different word tokens vectors of Q’, K’, V’ in probability space. The points that are closer to the lower right-hand corner in each graph are closer in probability space to the word vector “dog”. From this plot, it is obvious that the Boltzmann distributions of the individual vectors don’t overlap with the vector distribution for “dog”. Also, the distributions are divergent from each other. The vector distribution for “cat” is the closest to the vector distribution for “dog” in this plane.

The probability distribution generated using the Boltzmann Equation (31) was used to plot the distributions in the Bhattacharya coefficient vs. Jensen-Shannon divergence (JSD) plane in Figure 19. We used the JSD as the f-divergence measure for the distributions since this measure, unlike the Kullback-Liebler divergence, is symmetric. Also, JSD can be applied to distributions with arbitrary support. The JSD is a non-negative number upper bounded by 1 since in our study, we used base 2 for the logarithm to compute the JSD.

**Figure 20:**
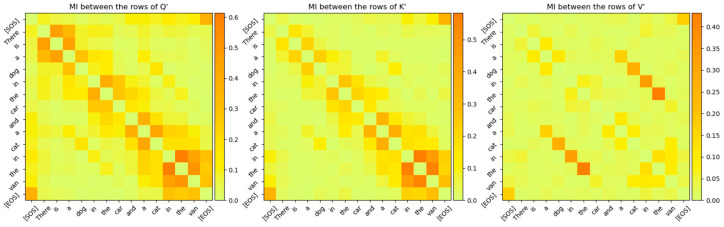
Mutual Information between the rows of $Q^{\prime },K^{\prime },V^{\prime }$ for Attention Layer 3. The relationships between the word sequences “There is a”, “in the car”, and “in the van” are clearly visible even though these vectors are projected to a high-dimensional vector space. The darker colored MIs are different compared to attention layer 0, indicating that this layer is focused on different relationships between words compared to attention layer 0. The diagonal entries, which are entropies of the token vectors, have been intentionally zeroed out so that the other MI values are visible.

The fact that each attention layer is focused on different relationships between the words of the sentence can be seen by comparing Figure 16 and Figure 20. The MI between the words of the sentence for attention layer 3 is shown in Figure 20. The larger values of the MI correspond to words that have a stronger relationship with each other. Comparing these MIs with the MIs of attention layer 0 shows that attention layer 3 is attending to a different part of the sentence.

**Figure 21:**
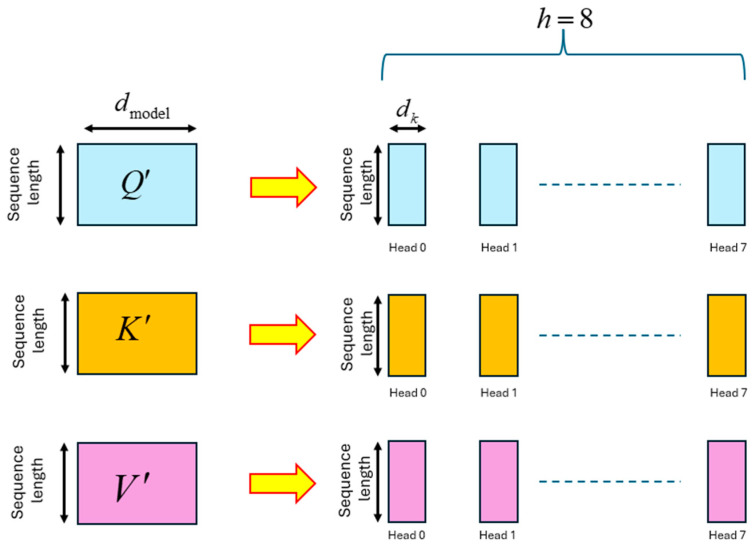
Partitioning of the Q’, K’, V’ matrices into h = 8 sub-matrices for multi-head processing.

The $Q^{\prime },K^{\prime },V^{\prime }$ matrices are partitioned into multiple sub-matrices, which are processed by different heads of the multi-head attention layers. The partitioning methodology is illustrated in Figure 21. The Transformer paper uses 8 heads for the multi-head processing operation.

Each of the heads formed from the $Q^{\prime },K^{\prime },V^{\prime }$ matrices is denoted as ${\tilde{Q}}_{i},{\tilde{K}}_{i},{\tilde{V}}_{i}$ for i=0,1,…7. These heads are each of dimension (*sequence_length* $\times \;d_{k}$), where $d_{k}=\frac{d_{\mathrm{model}}}{h}$. Since h=8 in the Transformer paper, the value of $d_{k}$ is 64. Each head ${\tilde{Q}}_{i}$ is multiplied by the transpose of the head ${\tilde{K}}_{i}$, and the product is scaled. The scaled product is sent through a softmax operation to obtain the attention scores matrix $A_{i}$. This is expressed as:

(32) $$A_{i}=\mathrm{softmax}\left(\frac{{\tilde{Q}}_{i}\times {\tilde{K}}_{i}^{T}}{\sqrt{d_{k}}}\right)\;\mathrm{for}\;i=0,1,\ldots h-1$$

The attention scores matrix $A_{i}$ has the dimensions (*sequence_length* × *sequence_length*). Each entry of this matrix signifies the linear relationship that the Transformer has learnt between the input words of the sentences. The softmax operation on a vector z of N elements generates probabilities for each element of z according to the following expression:

(33) $$\mathrm{softmax}\left(z\right)=\left[\frac{e^{z\left[0\right]}}{\sum_{i=0}^{N-1}e^{z\left[i\right]}},\frac{e^{z\left[1\right]}}{\sum_{i=0}^{N-1}e^{z\left[i\right]}},\ldots ,\frac{e^{z\left[N-1\right]}}{\sum_{i=0}^{N-1}e^{z\left[i\right]}}\right]$$

**Figure 22:**
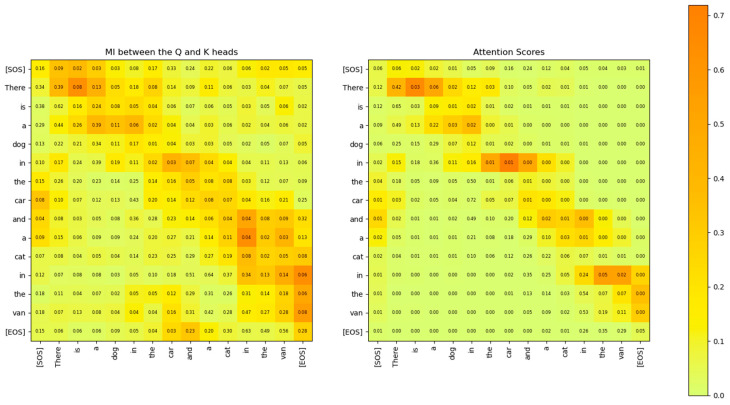
Comparison of the Mutual Information between the Q and K heads (**left**) and the attention scores (**right**) for attention layer 2, head 2. The two matrices show similar relationships between the words of the sentence. However, the MI matrix exposes weaker relationships that are not visible in the attention scores matrix.

We compare the attention scores with the MI between the rows of the ${\tilde{Q}}_{i}$ and ${\tilde{K}}_{i}$ matrices in Figure 22. This figure shows that the MI matrix highlights similar relationships between the words of the input sentence as the attention scores matrix. However, the MI matrix exposes additional weaker relationships between words that are not visible in the attention scores matrix.

**Figure 23:**
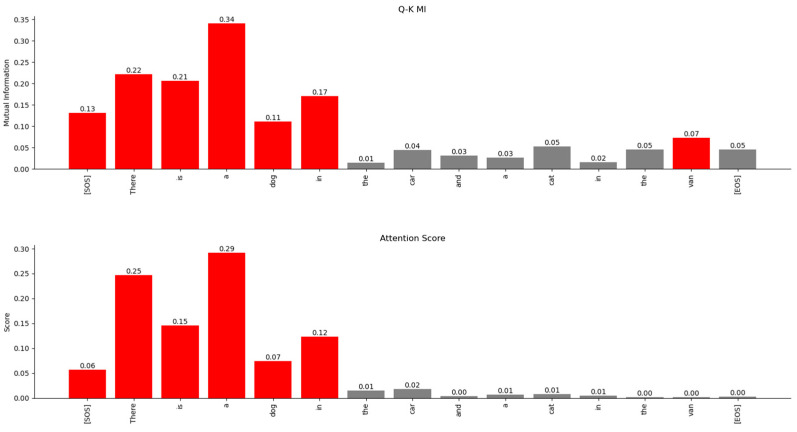
Relationship of the word ‘dog’ with other words in the sentence. The MI-based relationships (**top**) and the Attention Scores-based relationships (**bottom**) show similar trends. However, MI exposes a weaker relationship with the word “van”, which is not visible in the Attention Scores matrix.

**Figure 24:**
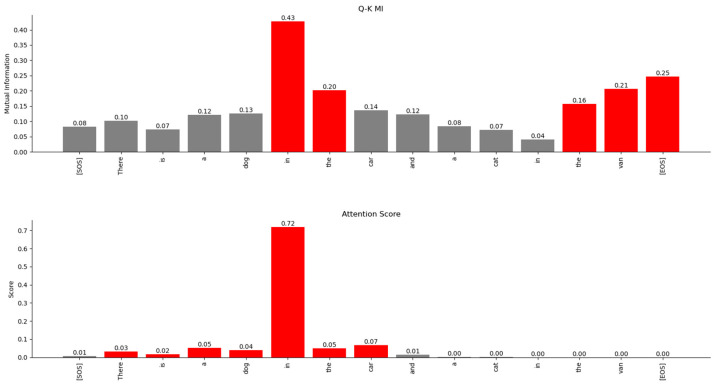
Relationship of the word ‘car’ with other words in the sentence. The MI-based relationships (**top**) and the Attention Scores-based relationships (**bottom**) show similar trends. However, MI exposes a weaker relationship with the word “van,” which is not visible in the Attention Scores matrix.

**Figure 25:**
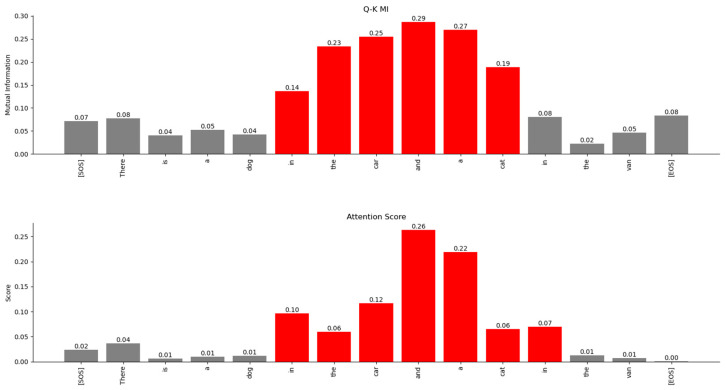
Relationship of the word ‘cat’ with other words in the sentence. The MI-based relationships (**top**) and the Attention Scores-based relationships (**bottom**) show similar trends.

The relationship between the words of the sentence from the MI matrix and the attention scores in Figure 22 is compared in Figure 23, Figure 24 and Figure 25. These figures show that the MI matrix and the attention score matrix exhibit a similar trend in the relationship between the words of the sentence, with the MI matrix exposing additional weaker relationships not visible in the attention score matrix.

The attention scores matrix for each head is multiplied by the corresponding ${\tilde{V}}_{i}$ matrix of that head to form a weighted value matrix as expressed below:

(34) $$\bar{\bar{V_{i}}}=A_{i}\times \tilde{V_{i}}$$

**Figure 26:**
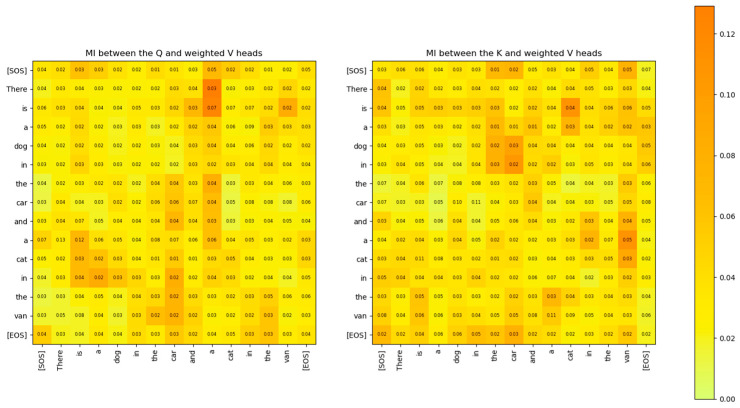
MI between Q head and weighted V head, and the MI between the K head and weighted V head for attention layer 2 and head 2. The MI shows how much information from Q and K heads is contained in the weighted V head matrix.

The dimensions of the matrix $\bar{\bar{V_{i}}}$ are (*sequence_length* $\times \;d_{k}$). The matrix $\bar{\bar{V_{i}}}$ represents the proportionate mixing of the encoded vectors from the ${\tilde{Q}}_{i}$ and ${\tilde{K}}_{i}$ matrices based on the attention scores. The amount of information from the ${\tilde{Q}}_{i}$ and ${\tilde{K}}_{i}$ matrices contained in $\bar{\bar{V_{i}}}$ is shown in Figure 26.

**Figure 27:**
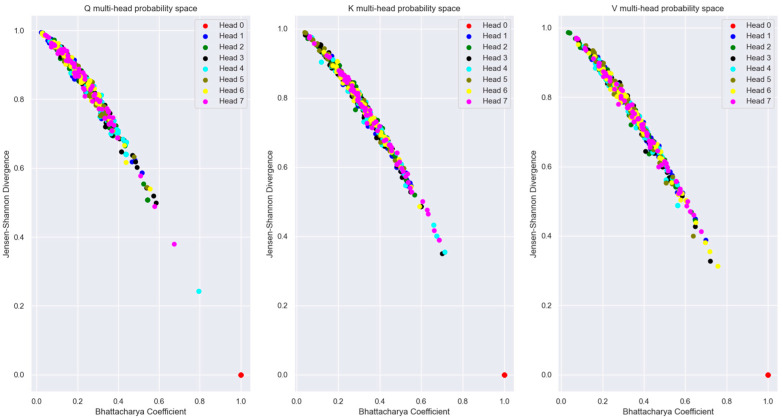
Distribution of the same word vector coordinates in different attention layer heads with respect to head 0 of the encoder. The plots show that the Boltzmann distributions for the same words are sufficiently separated statistically between the different attention heads so that each head can attend to different latent parameters of the words. Several words from the validation dataset are plotted in this probability distribution plane.

During training, the Transformer learns the projection matrices $W_{Q},W_{K,}W_{V}$ to ensure that words that are unrelated to each other in the sentence have the maximum distance between their distributions. Also, the projection matrices ensure that distributions of the same word vectors in different attention heads are statistically as far apart as possible so that each attention head can attend to and learn different latent parameters of the input words. The distribution of the same words across multiple attention heads is shown in Figure 27. From this figure, it is evident that the Transformer learns the projection matrix weights to sufficiently separate the distributions of the same word vector across multiple attention heads.

**Figure 28:**
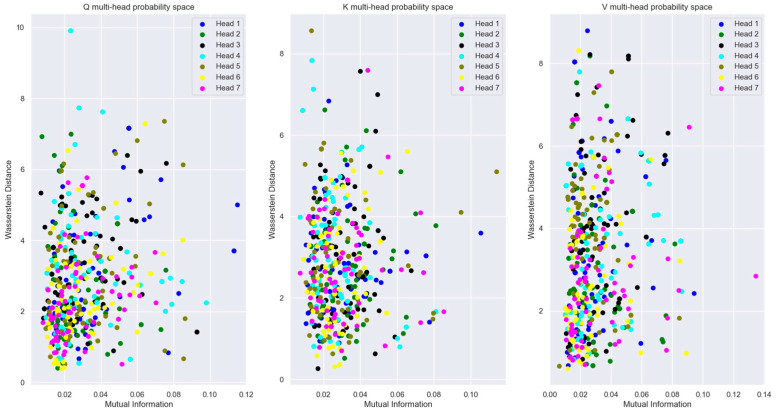
Plots of the same word vectors across multiple attention heads with reference to head 0. The plot shows that the coordinate distributions of the word vectors are not statistically far apart. However, the Mutual Information between the same word vectors across multiple attention heads is very low, making them mutually independent. Several words from the validation dataset are plotted in this figure.

The plot of Mutual Information vs. Wasserstein distance for the same word vector coordinate distribution across different attention heads (with respect to head 0) in Figure 28 highlights the fact that even though the coordinate distributions are not far from each other, the MI is low across the attention heads.

To determine if the points in Figure 28 form specific distribution clusters we used a modified Spectral Clustering algorithm [19] to identify these clusters. The first step of the Spectral Clustering algorithm is to create a similarity or affinity graph using an adjacency matrix A. Each element $a\left(x_{i},x_{j}\right)$ of this matrix is a pairwise affinity value between the samples of the distribution and is expressed as:

(35) $$a\left(x_{i},x_{j}\right)=\exp\left(-\beta d\left(x_{i},x_{j}\right)\right)\;\mathrm{where}\;d\left(x_{i},x_{j}\right)\;\mathrm{is}\;\mathrm{the}\;\mathrm{statistical}\;\mathrm{distance}$$

The statistical distance in our Spectral clustering algorithm is a function of the Wasserstein distance and the Bhattacharya coefficient. We used a value of β=7.5 for the decay rate of the above exponential to ensure that points which are far away from each other are grouped in different clusters. The degree matrix D is formed by summing the rows of the adjacency matrix and forming a diagonal matrix from each row sum. The graph Laplacian was created using the following expression:

(36) $$L=I_{n}-D^{-1/2}AD^{-1/2}$$

**Figure 29:**
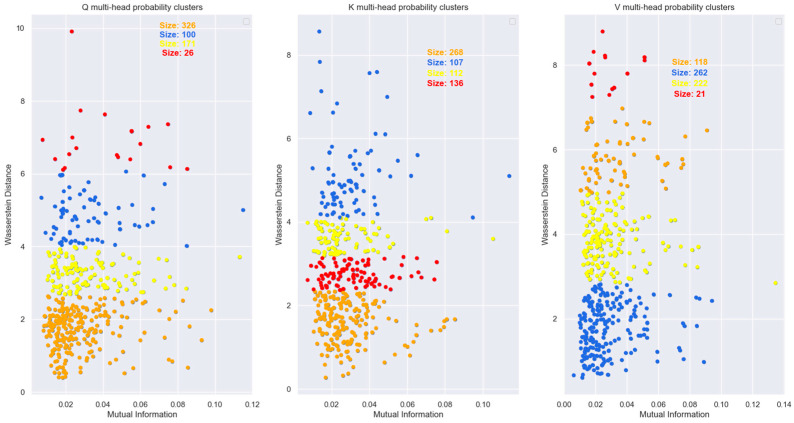
Spectral clustering of the data points from Figure 28. The clusters show that most of the points in the plane lie in a cluster with statistically similar coordinate distributions. However, the distribution of the same word vectors across the various heads are mutually independent.

The Laplacian matrix was decomposed as:

(37) $$L=U\Lambda U^{T}$$

where U contains the eigenvectors of the Laplacian and Λ the eigenvalues. We used a K-means algorithm on the rows of the U matrix corresponding to the k smallest eigenvalues to group the data points into clusters. The output of the Spectral Clustering algorithm is shown in Figure 29.

### 3.4. Upper Bound on the Number of Attention Heads Based on the Supermodularity of Mutual Information for Independent Heads

From the results in the previous section, it is evident that during training, the Transformer learns the coefficients of the projection matrices to ensure that distributions of the same word vectors in different attention heads are mutually independent. This ensures that each attention head can learn different latent parameters of the input words. We can use the property of supermodularity of mutual information to place an upper bound on the number of heads of the Transformer model.

For a set Ω and sets X and Y such that ∀X⊆Y⊆Ω, then a function $f:2^{\Omega }\to \mathbb{R}$ is supermodular if ∀z∉Y we have:

(38) $$f\left(Y\cup \left\{z\right\}\right)-f\left(Y\right)\geq f\left(X\cup \left\{z\right\}\right)-f\left(X\right)$$

This definition of supermodularity implies that if f is a utility function, then the utility gain from adding a new element $\left\{z\right\}$ to the larger set Y is more than adding it to the smaller set X.

Let $S,X_{1},X_{2},Y$ be random variables such that $X_{1}$ is mutually independent of the random variables $\left(S,X_{2}\right)$. Then from [20,21] we have the following inequality and its proof:

(39) $$I\left(Y;S,X_{1},X_{2}\right)-I\left(Y;S,X_{2}\right)\geq I\left(Y;S,X_{1}\right)-I\left(Y;S\right)$$

This can be seen as the supermodularity property of mutual information under independence. The proof relies on the following properties of mutual information [22]:

(40) $$\begin{array}{l} I\left(Y;X\left|Z\right.\right)=I\left(X;Y\left|Z\right.\right)\;\;\left(\mathrm{Symmetry}\;\mathrm{of}\;\mathrm{conditional}\;\mathrm{MI}\right)\\ I\left(Y;X,Z\right)=I\left(Y;Z\right)+I\left(Y;X\left|Z\right.\right)=I\left(Y;X\right)+I\left(Y;Z\left|X\right.\right)\;\;\left(\mathrm{Chain}\;\mathrm{rule}\;\mathrm{of}\;\mathrm{MI}\right) \end{array}$$

**Proof.**  (41) $$\begin{array}{l} I\left(Y;S,X_{1},X_{2}\right)-I\left(Y;S,X_{2}\right)\\ =I\left(Y;S,X_{2}\right)-I\left(Y;X_{1}\left|S,X_{2}\right.\right)-I\left(Y;S,X_{2}\right)\;\left(\mathrm{chain}\;\mathrm{rule}\right)\\ =I\left(Y;X_{1}\left|S,X_{2}\right.\right)\\ =I\left(X_{1};Y\left|S\right.,X_{2}\right)\;\left(\mathrm{by}\;\mathrm{symmetry}\;\mathrm{property}\right)\\ =I\left(X_{1};Y,S,X_{2}\right)-I\left(X_{1}\left|S,X_{2}\right.\right)\;\left(\mathrm{chain}\;\mathrm{rule}\right)\\ =I\left(X_{1};Y,S,X_{2}\right)\;\left(\mathrm{since}\;X_{1}⊥⊥\left(S,X_{2}\right)\right)\\ =I\left(Y,S,X_{2};X_{1}\right)\;\left(\mathrm{symmetry}\right)\\ \geq I\left(Y,S;X_{1}\right)\;\left(\mathrm{monotonicity}\;\mathrm{of}\;\mathrm{MI}\right)\\ =I\left(X_{1};Y,S\right)\;\left(\mathrm{symmetry}\right)\\ =I\left(X_{1};S\right)+I\left(X_{1};Y\left|S\right.\right)\;\left(\mathrm{chain}\;\mathrm{rule}\right)\\ =I\left(X_{1};Y\left|S\right.\right)\;\left(\mathrm{since}\;X_{1}⊥S\right)\\ =I\left(Y;X_{1}\left|S\right.\right)\;\left(\mathrm{symmetry}\right)\\ =I\left(Y;S,X_{1}\right)-I\left(Y;S\right)\;\left(\mathrm{chain}\;\mathrm{rule}\right)\\ \;\;\;\;\;\;\;\;□ \end{array}$$

The inequality in (39) can also be verified using the AITIP tool described in [23].

If the different attention heads of a specific encoder or decoder layer is denoted by the random variables $S,X_{1},X_{2},Y$ then we can numerically compute the multivariate mutual information: $I\left(Y;S,X_{1},X_{2}\right),I\left(Y;S,X_{2}\right),I\left(Y;S,X_{1}\right),I\left(Y;S\right)$ and check if (39) is satisfied for the attention heads. If this inequality is satisfied, then we can conclude that the distributions of the same word vector encoding across multiple attention heads are independent and the number of heads used in the Transformer model satisfies the supermodularity constraints. This implies that the number of attention heads in the encoder and decoder can be upper bounded by the inequality in (39).

To compute each term of this inequality we used the following algebraic manipulation:

(42) $$I\left(X;Y,Z\right)=I\left(X;Y\right)+I\left(X;Z\left|Y\right.\right)\;\left(\mathrm{from}\;\mathrm{chain}\;\mathrm{rule}\right)$$

The first term of the above equation can be numerically computed using (10). The second term (i.e., the conditional probability term) can be computed as follows:

(43) $$I\left(X;Z\left|Y\right.\right)=\sum_{x\in X}\sum_{y\in Y}\sum_{z\in Z}p\left(x,y,z\right)\log\left[\frac{p\left(x,z\left|y\right.\right)}{p\left(x\left|y\right.\right)p\left(z\left|y\right.\right)}\right]$$

Since, $p\left(x,z\left|y\right.\right)=\frac{p\left(x,y,z\right)}{p\left(y\right)}$ we can write the above expression as:

(44) $$\begin{array}{c} I\left(X;Z\left|Y\right.\right)=\sum_{x\in X}\sum_{y\in Y}\sum_{z\in Z}p\left(x,y,z\right)\log\left[\frac{p\left(x,y,z\right)}{p\left(x\left|y\right.\right)p\left(z\left|y\right.\right)p\left(y\right)}\right]\\ \;\;\;=\sum_{x\in X}\sum_{y\in Y}\sum_{z\in Z}p\left(x,y,z\right)\log\left[\frac{p\left(x,y,z\right)}{p\left(x,y\right)p\left(z\left|y\right.\right)}\right]\\ \;\;\;\;\;\;=\sum_{x\in X}\sum_{y\in Y}\sum_{z\in Z}p\left(x,y,z\right)\log\left[\frac{p\left(x,y,z\right)}{p\left(x,y\right)\left(p\left(y,z\right)/p\left(y\right)\right)}\right] \end{array}$$

To compute the mutual information involving four random variables we used the following algebraic manipulation:

(45) $$I\left(W;X,Y,Z\right)=I\left(W;X,Y\right)+I\left(W;Z\left|X,Y\right.\right)\;\left(\mathrm{from}\;\mathrm{chain}\;\mathrm{rule}\right)$$

The first term in the previous equation can be numerically computed using (42)–(44). The second term (i.e., the conditional probability term) can be computed as follows:

(46) $$I\left(W;Z\left|X,Y\right.\right)=\sum_{w\in W}\sum_{x\in X}\sum_{y\in Y}\sum_{z\in Z}p\left(w,x,y,z\right)\log\left[\frac{p\left(w,z\left|x,y\right.\right)}{p\left(w\left|x,y\right.\right)p\left(z\left|x,y\right.\right)}\right]$$

Since, $p\left(w,z\left|x,y\right.\right)=\frac{p\left(w,x,y,z\right)}{p\left(x,y\right)}$ we can write the above expression as:

(47) $$\begin{array}{c} I\left(W;Z\left|X,Y\right.\right)=\sum_{w\in W}\sum_{x\in X}\sum_{y\in Y}\sum_{z\in Z}p\left(w,x,y,z\right)\log\left[\frac{p\left(w,x,y,z\right)}{p\left(w\left|x,y\right.\right)p\left(z\left|x,y\right.\right)p\left(x,y\right)}\right]\\ \;\;\;=\sum_{w\in W}\sum_{x\in X}\sum_{y\in Y}\sum_{z\in Z}p\left(w,x,y,z\right)\log\left[\frac{p\left(w,x,y,z\right)}{p\left(w,x,y\right)p\left(z\left|x,y\right.\right)}\right]\\ \;\;\;\;\;\;\;=\sum_{w\in W}\sum_{x\in X}\sum_{y\in Y}\sum_{z\in Z}p\left(w,x,y,z\right)\log\left[\frac{p\left(w,x,y,z\right)}{p\left(w,x,y\right)\left(p\left(x,y,z\right)/p\left(x,y\right)\right)}\right] \end{array}$$

**Figure 30:**
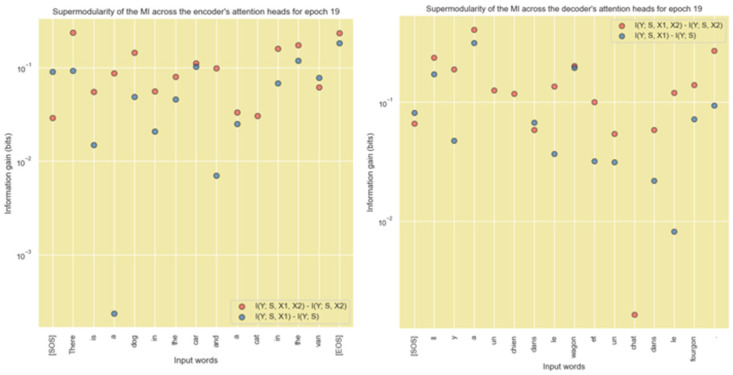
Numerical verification of the supermodular property of mutual information for the encoder’s (left plot) and decoder’s (right plot) heads shows that the Transformer’s attention heads satisfy the inequality $I\left(Y;S,X_{1},X_{2}\right)-I\left(Y;S,X_{2}\right)\geq I\left(Y;S,X_{1}\right)-I\left(Y;S\right)$. This implies that the distributions of the heads are mutually independent. The supermodularity condition can be used as an upper bound for the number of attention heads. In other words, the number of attention heads cannot exceed a threshold that will violate the supermodularity constraint.

The results of the numerical computation of the supermodular inequality are shown in Figure 30. This figure clearly shows that if the different attention heads of a specific encoder or decoder layer is denoted by the random variables $S,X_{1},X_{2},Y$ then on average the information gain $I\left(Y;S,X_{1},X_{2}\right)-I\left(Y;S,X_{2}\right)$ is greater than or equal to $I\left(Y;S,X_{1}\right)-I\left(Y;S\right)$ for every word vector distribution across the attention heads. This implies that the distribution of the encoded word vector is independent across the heads. The supermodularity condition can therefore be used to upper bound the number of heads of the attention layer.

### 3.5. Relationship Between the Encoder’s K and V Matrix Output to the Decoder’s Cross-Attention Layer’s Q Matrix Input

Each of the $\bar{\bar{V_{i}}}$ matrices is concatenated to form the multi-head attention matrix M of dimension (*sequence_length* $\times \;d_{\mathrm{model}}$). This concatenated matrix is then projected along the column space of an output projection matrix $W_{O}$ of dimension ($d_{\mathrm{model}}\times d_{\mathrm{model}}$) to generate a multi-head attention output matrix $M^{\prime }$ of dimension (*sequence_length* $\times \;d_{\mathrm{model}}$) as expressed below:

(48) $$M\times W_{O}=M^{\prime }$$

The matrix $M^{\prime }$ is sent through a feed-forward network which performs the following operation with weight matrices $W_{1}$ and $W_{2}$ and bias vectors $b_{1}$ and $b_{2}$ which are learned during the training phase:

(49) $$FF\left(x\right)=\left[ReLU\left(xW_{1}+b_{1}\right)\right]W_{2}+b_{2}$$

To quantify the amount of information that is passed from the input to the output of the feedforward block, we viewed this block as a Shannon information channel and estimated the conditional probability $P\left(y\left|m^{\prime }\right.\right)$ between the input and output symbols of this channel quantized to 100 different values (i.e., 10,000 input and output combinations of discrete symbols). Using this, we computed the capacity of this channel using the Blahut-Arimoto Algorithm, and that the capacity of this channel is about 3 bits. In other words, only about 3 bits of information from the input symbol to the output symbol can pass through this channel unaltered. The output matrix of the feedforward block has the dimension (*sequence_length* $\times \;d_{\mathrm{model}}$). This output is normalized and sent to the input of the next sublayer of the encoder. The output of the 6th (i.e., last) encoder sublayer is sent to the value and key inputs of the cross multi-head attention block of the decoder.

**Figure 31:**
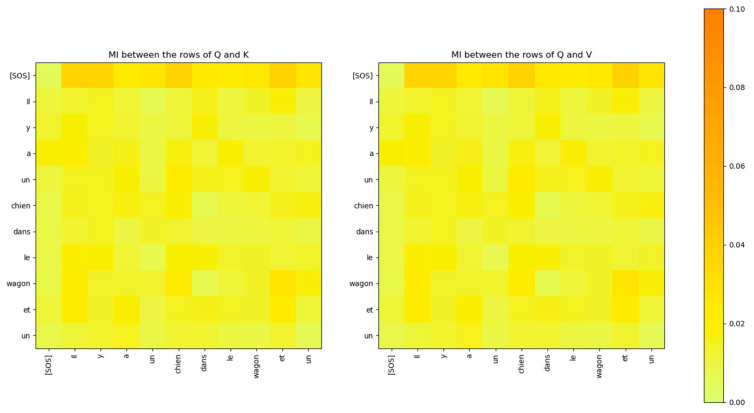
The Mutual Information between the rows of the input Q matrix of the cross-attention layer of the decoder and the rows of the input K and V matrices from the encoder is low.

The output of the masked multi-head attention sub-layer of the decoder is sent to the Q input of the cross multi-head attention sub-layer. The K and V inputs to this block come from the Encoder. Figure 31 shows the MI between the rows of the Q matrix and the rows of the K and V matrices. It is evident that the rows of the Q matrix are independent of the rows of the K and V matrices.

**Figure 32:**
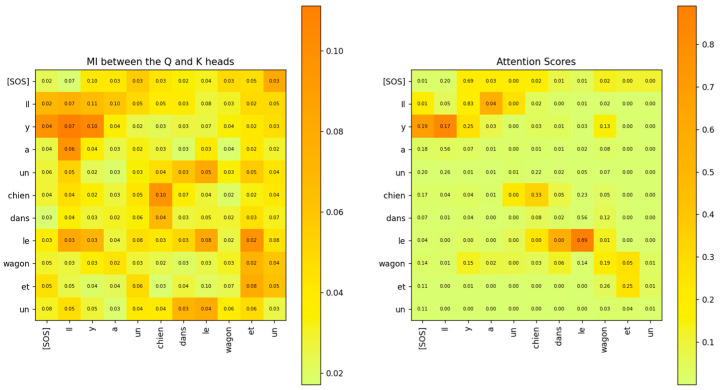
Comparison of the Mutual Information between the Q and K heads (**left**) and the attention scores (**right**) for attention layer 2, head 2 of the decoder. The two matrices show similar relationships between the words of the sentence. However, the MI matrix exposes weaker relationships that are not visible in the attention scores matrix.

The MI between the rows of the Q and K attention heads of the decoder layer 2 is compared to the attention scores in Figure 32. The figure shows that the MI highlights a similar relationship between the words of the decoder and the attention scores. However, the MI exposes additional weaker relationships that are not visible in the attention scores matrix.

### 3.6. Projection Layer

**Figure 33:**
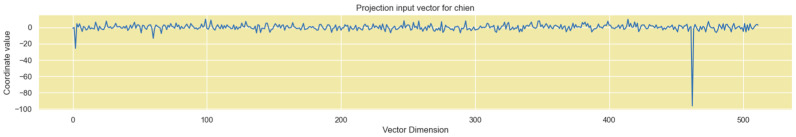
Projection input vector has a single high coordinate value in dimension 461. All other dimension coordinates have low values.

The decoder output is multiplied by a linear projection matrix, and the result is sent to a softmax block, which outputs the probability for each word in the vocabulary. The word with the largest probability is output as the next word in the output sequence of the decoder. We analyzed the decoder output vector, which is input to the projection layer. It is a vector of length $d_{\mathrm{model}}$ during the probing phase. The plot of the coordinate value of this vector for a specific word is shown in Figure 33. Except for one dimension, all other dimensions have low coordinate values.

**Figure 34:**
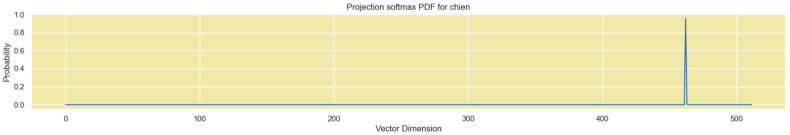
Boltzmann probability distribution of the Projection vector input.

The corresponding Boltzmann probability distribution in Figure 34 shows a single dimension with a high value.

**Figure 35:**
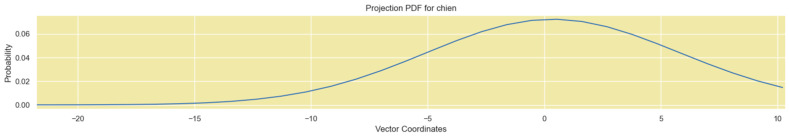
The distribution of the other coordinate values of the Projection vector has a Gaussian shape.

The distribution of the coordinates in the other dimensions has a Gaussian shape, with a mean at a coordinate value close to 1.0, as shown in Figure 35. The coordinates are distributed over the range $\left[-100,10\right]$.

These figures show that the decoder output probability is concentrated on a single dimension of the multidimensional vector. This dimension corresponds to the word that the Transformer decodes in response to the input. This input vector is multiplied by a projection matrix, and the output is provided to the softmax block to generate the probabilities for each word of the vocabulary. The word with the highest probability is output as the next decoded word by the Transformer. This word is then concatenated with all the previous words in the decoded sequence and fed as input to the Transformer to decode the next word.

## 4. Troubleshooting Learning Problems in the Transformer Model

The Information-Theoretic techniques that we used to analyze the Transformer’s encoding of the relationship between word vectors in high-dimensional space can be used for troubleshooting performance issues within the layers of the Transformer. To create an impaired Transformer model, we lowered the dimension of the model to $d_{\mathrm{model}}=32$ from $d_{\mathrm{model}}=512$ while keeping everything else the same in the model. We trained this model on the same training set as the original model. This model had a BLEU score of about 0.2 compared to a BLEU score of 0.45 with the original model.

**Figure 36:**
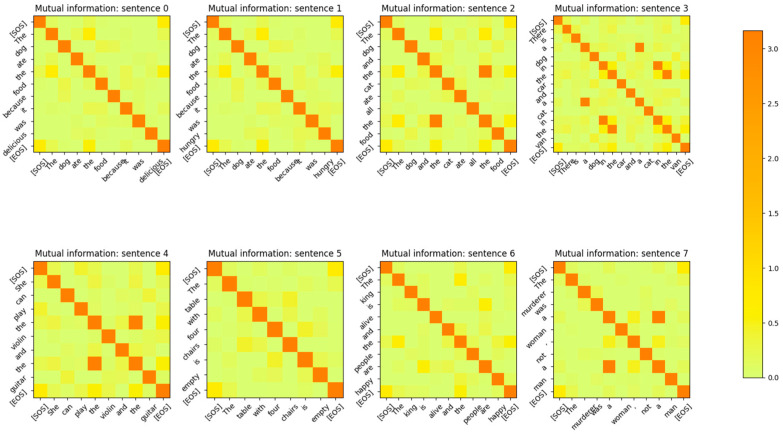
Mutual Information between the Embedding output vectors for $d_{\mathrm{model}}=32$. Embedding vectors for different tokens are not completely mutually independent.

The first thing that we noticed with the impaired model is that the MI between the Input Embedding layer output vectors was significantly higher. In other words, the Embedding layer neural network was not able to learn weights that could generate mutually independent vectors for the input word tokens. A comparison of the MI of the embedding vectors of Figure 36 with Figure 3 clearly shows that if the dimension of the model is not sufficiently large, the embedding vectors are likely to have a high MI between them. Therefore, one way to check for impaired performance in the embedding layer is to inspect the MI between the embedding vectors.

**Figure 37:**
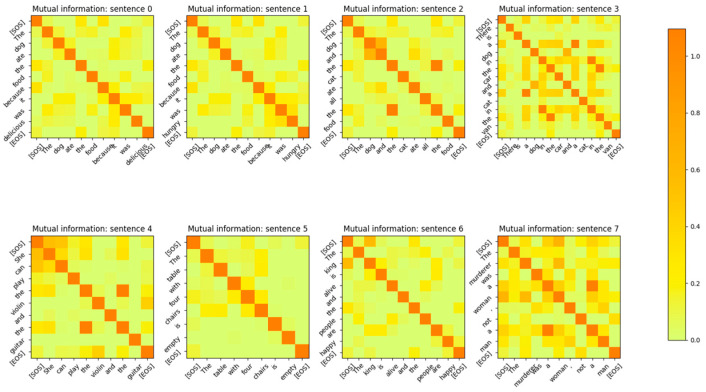
Mutual Information between the Input Embedding output vectors for $d_{\mathrm{model}}=8$. The embedding vectors for different tokens are not mutually independent.

Reduction of the dimension of the model further to $d_{\mathrm{model}}=8$ resulted in even higher MI values between embedding vectors as shown in Figure 37. This indicates that as the model dimension increases, the Input Embedding layer of the Transformer is able to learn weights to generate mutually independent embedding vectors.

**Figure 38:**
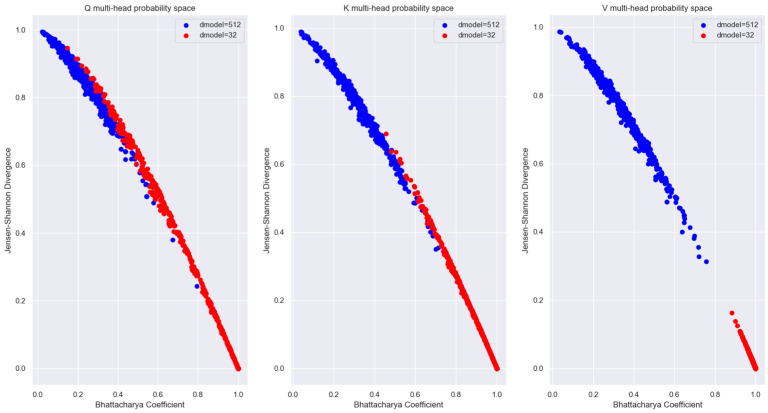
Comparison of the statistical separation of vector coordinate distribution for the same word vector across multiple attention heads for $d_{\mathrm{model}}=512$ and $d_{\mathrm{model}}=32$. The statistical separation is smaller with $d_{\mathrm{model}}=32$.

Figure 27, Figure 28 and Figure 29 showed that for a sufficiently large model dimension, there is a sufficient statistical distance between the distribution of the word vectors across multiple attention heads of the encoder and the decoder. However, if the Transformer model is impaired, the statistical distance between the vectors of the same word across multiple heads becomes smaller. In other words, the Transformer is unable to learn values of the projection matrices that statistically separate the vector coordinate distribution across multiple attention heads. This behavior is evident in Figure 38. Therefore, statistical separation of the vector coordinate distribution across attention heads is a measure that can be used to troubleshoot learning problems with the Transformer model.

**Figure 39:**
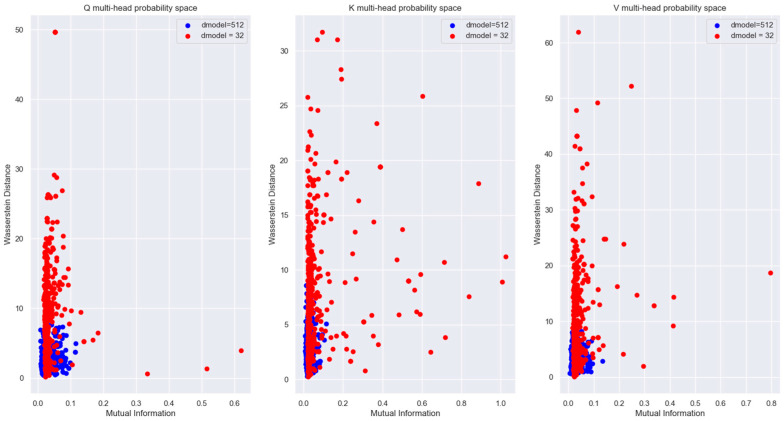
Comparison of the MI and Wasserstein distance between the vector coordinate distribution across multiple attention heads for $d_{\mathrm{model}}=512$ and $d_{\mathrm{model}}=32$. The figures show that the MI between the distribution across multiple attention heads is reduced with the reduced dimension model. Also, the distance between the coordinate distributions increased with the reduced dimension model.

Figure 39, shows that the MI between the vector coordinate distribution across multiple attention heads becomes smaller as the model dimension reduces. This behavior is seen for the attention heads of the encoder and the decoder. Therefore, to troubleshoot Transformer performance problems, viewing the distribution of the vector coordinates in an Information-Theoretic plane can help identify learning bottlenecks in the different layers of the Transformer.

**Figure 40:**
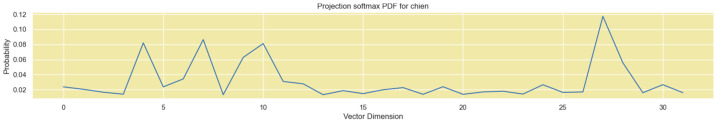
Boltzmann probability distribution of the Projection vector input for $d_{\mathrm{model}}=32$. The impaired model shows that there are several dimensions with relatively high probability values, which will reduce the confidence of the softmax probabilities at the projection layer output.

The Boltzmann distribution of the projection vector output for the impaired Transformer model shown in Figure 40 highlights another way to troubleshoot learning problems with the Transformer. Comparing this figure with Figure 34, it is evident that the input vector of the Projection layer does not have a clear winner for decoding the next word in the impaired Transformer model. The Boltzmann probability distribution has several peaks, which adversely affect the performance of the Transformer.

## 5. Analysis of the Transformer Vectors Using Information Geometry

As we saw in the previous sections, the word tokens in each layer of the Transformer are encoded in a high-dimensional vector space, which makes it very difficult to visualize the relationships between words. However, to understand the underlying mechanism of the Transformer we need to glean a perspective of this high-dimensional vector space. In the previous sections, our analysis was limited to a Euclidean space. However, in this section, we consider the distribution of the high-dimensional word vectors on a statistical manifold and analyze the relationships between them on this manifold. In Section 3, we showed how to derive a probability distribution of the energy assigned to each of the dimensions of the vectors using the Boltzmann Equation (31). We can view these probability distributions as points on a high-dimensional statistical manifold called a Riemann manifold and use techniques from Information Geometry [24] to analyze the relationship between these vectors. Consider a probability space $\left(\Omega ,F,P\right)$ consisting of a set Ω of all possible outcomes (i.e., the sample space), a σ—field F of subsets of Ω and a probability measure P on $\left(\Omega ,F\right)$. The push-forward measure of P by the random variable X:Ω→X is denoted as $X_{*}P$. We assume that $X_{*}P$ is continuous with respect to a measure μ. For a discrete random variable X, the measure μ is a counting measure; for a continuous random variable, the measure μ is a Lebesgue measure. The Radon-Nikodym derivative $d\left(X_{*}P\right)/d\mu :$X→ℝ can be considered as a probability mass function (PMF) for the discrete case or a probability density function for the continuous case, and is denoted as $p\left(x\right)$.

**Figure 41:**
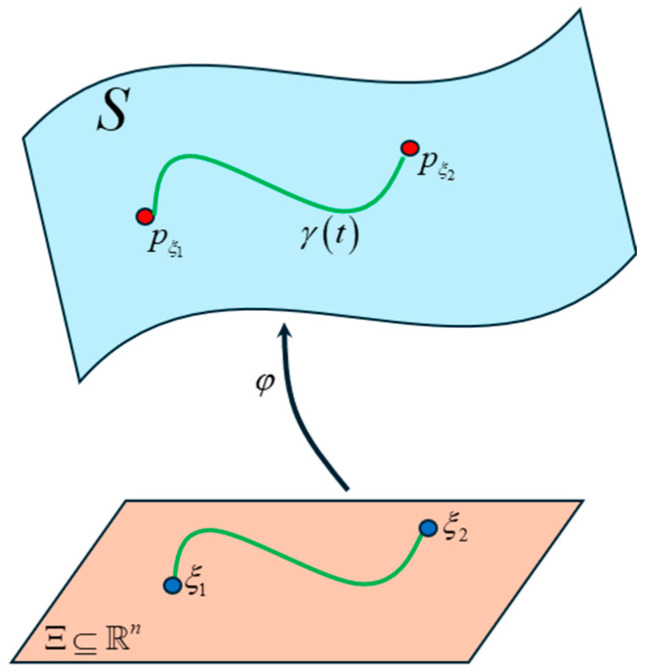
Statistical manifold where each point is a probability distribution of the Transformer word vectors. Our goal is to find the shortest curve (i.e., geodesic) that connects these points.

Consider a statistical manifold S such that the points on this manifold are probability distributions parameterized by n—dimensional vectors $\xi =\left(\xi_{1},\xi_{2},\ldots \xi_{n}\right)$. Each of the distributions are denoted as $p_{\xi }$. This is illustrated in Figure 41. In this figure, the parameter $\xi \in \Xi \subseteq {\mathbb{R}}^{n}$ and the mapping $\xi \to p_{\xi }$ is injective and is denoted by φ. With respect to the Transformer model, each of the points on the manifold are distributions of the high-dimensional word vectors.

The assumption is that ξ:Ξ→ℝ is $C^{\infty }$ so that we can take derivatives of $p\left(x;\xi \right)$ with respect to the parameters. Defining the operator $\partial_{i}\equiv \frac{\partial }{\partial \xi_{i}}$ we can express the derivative at each point on the manifold as $\partial_{i}p\left(x;\xi \right)$ or $\partial_{i}p_{\xi }$. We induce a differential structure to the manifold S by assuming that $\left\{\partial_{1}p_{\xi },\ldots ,\partial_{n}p_{\xi }\right\}$ are linearly independent, the mapping $\varphi \left(\xi \right)=p_{\xi }$ is a homeomorphism on its image and the partial derivatives $\partial_{i}p_{\xi }\left(x\right)$ commute with the integrals so that we can write $\int dx\partial_{i}p_{\xi }\left(x\right)=\partial_{i}\int dxp_{\xi }\left(x\right)=\partial_{i}1=0$.

To use techniques [25] from Information Geometry, we convert this statistical manifold into a Riemannian manifold by introducing a metric on it. To do this, we consider the n×n Fisher information matrix $G\left(\xi \right)$ at a point $p_{\xi }$ on the manifold. For 1≤i,j≤n, each of the elements of this matrix is denoted by $g_{ij}\left(\xi \right)$, which is defined as:

(50) $$g_{ij}\left(\xi \right)\equiv E_{\xi }\left[\partial_{i}l_{\xi }\partial_{j}l_{\xi }\right]\;\mathrm{where}\;l_{\xi }=\log p_{\xi }$$

Therefore, each element can be expressed as:

(51) $$g_{ij}\left(\xi \right)=\int_{X}p_{\xi }\left(\frac{\partial }{\partial \xi^{i}}\log p_{\xi }\right)\left(\frac{\partial }{\partial \xi^{j}}\log p_{\xi }\right)d\mu$$

The KL divergence between a probability distribution $p\left(x;\xi \right)$ and $p\left(x;\xi +\Delta \right)$ where Δ is an infinitesimal change in the parameter ξ, gives us [26] the same expression (up to a scaling factor) as (51). This is the reason that the Fisher metric $g_{\xi }$ is considered as a candidate metric in a statistical manifold. The Fisher metric is invariant under re-parametrizations of the sample space X [27] and is the unique Riemannian metric in statistical manifolds that is invariant under sufficient statistics [28]. It is for this reason that the Fisher metric is the preferred metric in Riemannian manifolds. Since the Fisher information matrix is symmetric and positive-definite it implies that $g_{ij}\left(\xi \right)=g_{ji}\left(\xi \right)$ is consistent with the requirement that $\left\{\partial_{1}p_{\xi },\ldots ,\partial_{n}p_{\xi }\right\}$ are linearly independent functions on X. The elements of the Fisher matrix can be expressed as [27]:

(52) $$g_{ij}\left(\xi \right)=-E\left[\partial_{j}\partial_{i}l\left(\xi \right)\right]$$

This form of the Fisher metric shows how points are mapped in S to their inner products on the tangent space $T_{p}S$ described in the following paragraph. This property of the Fisher metric induces the notion of length and angle between vectors on the tangent space of the Riemann manifold.

The curve $\gamma \left(t\right)$ in Figure 41 connects the two points on the manifold. The parameter t lies in an interval I⊂ℝ and it allows us to describe the position at any point in the curve. The goal of our analysis is to find the shortest curve or the geodesic on the manifold that connects the two points. Since each point on the manifold represents a probability distribution of a word, we can infer the relationship between the words that the Transformer learns during the training phase by computing the length of the geodesic between a reference point and the other points on the manifold.

**Figure 42:**
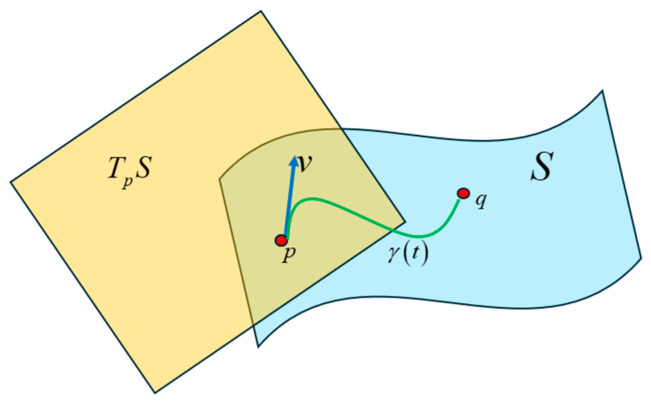
A tangent space defined at the point p on the manifold and the tangent vector v lies in this space. This vector is tangent to the curve at point p.

Unlike the Euclidean space, we cannot define a position vector on the manifold to find the distance between points. To calculate the length of the curve, we need to define a tangent space at the point p on the manifold and assign a tangent vector to the curve at this point. The tangent space $T_{p}S$ at point p on the manifold S is shown in Figure 42. This figure appears to imply that the tangent space and the vector that lies on it are outside of the manifold, implying that the manifold is embedded in a high-dimensional space. However, it is not necessary for a manifold to be embedded in a higher-dimensional space (i.e., the manifold is the entire space and there is nothing outside of it), in which case the tangent space drawn in Figure 42 is just an abstract mathematical concept to help visualize the space of tangent vectors.

To determine the length of a curve on the Riemannian manifold, the tangent vector at each point in the curve is computed by obtaining the derivative at that point. The geodesic between two points on the manifold is the curve $\gamma \left(t\right)$ that parallel transports its tangent vector along itself. The Fisher-Rao distance is the length of the geodesic between two points (i.e., probability distributions) on the same statistical manifold. Consider a curve ξ in the parameter space in the interval $\left[a,b\right]\in \mathbb{R}$. The image of this curve on the Riemann manifold is $\gamma \left(t\right)$ which can be obtained by the composite of the mapping functions: $\gamma \left(t\right)=\left(\varphi \circ \xi \right)\left(t\right)$ for $t\in \left[a,b\right]$. Since the Fisher metric endows the Riemann manifold with a vector dot-product operation, the length of $\gamma \left(t\right)$ can be computed as:

(53) $$l\left(\gamma \right)=\int_{a}^{b}\sqrt{{\langle \dot{\gamma }\left(t\right),\dot{\gamma }\left(t\right)\rangle }_{G\left(\xi \left(t\right)\right)}}dt=\int_{a}^{b}\sqrt{{\dot{\xi }}^{T}\left(t\right)G\left(\xi \left(t\right)\right)\dot{\xi }\left(t\right)}dt$$

where $\dot{\gamma }\left(t\right)=d\varphi_{\xi \left(t\right)}\left(\dot{\xi }\left(t\right)\right)$. The Fisher-Rao distance between two probability distributions $p_{\xi_{1}}$ and $p_{\xi_{2}}$ in the manifold S is the smallest length of the piecewise differentiable curves γ joining these two points. The geodesic $\gamma \left(t\right)=\left(\varphi \circ \xi \right)\left(t\right)$ can be obtained by solving for $\xi \left(t\right)$ in local coordinates the following geodesic differential equation:

(54) $${\ddot{\xi }}^{k}\left(t\right)+\sum_{i=1}^{n}\sum_{j=1}^{n}\Gamma_{ij}^{k}{\dot{\xi }}^{i}\left(t\right){\dot{\xi }}^{j}\left(t\right)=0,\;k\in \left\{1,\ldots ,n\right\}$$

where $\Gamma_{ij}^{k}$ are Christoffel symbols of the second kind. The values of the symbols can be obtained from the following equation:

(55) $$\sum_{k=1}^{n}g_{lk}\Gamma_{ij}^{k}=\frac{1}{2}\left(\frac{\partial }{\partial \xi^{i}}g_{jl}+\frac{\partial }{\partial \xi^{j}}g_{li}+\frac{\partial }{\partial \xi^{l}}g_{ij}\right),\;i,j,l\in \left\{1,\ldots ,n\right\}$$

**Figure 43:**
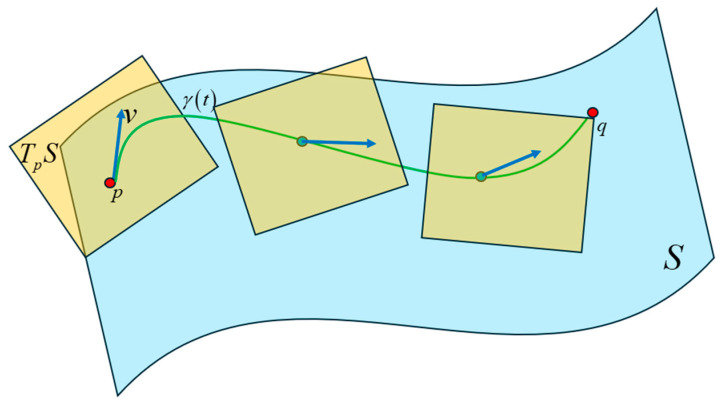
A tangent space is defined at each point on the curve on the Riemann manifold by taking the derivative at that point. The tangent vector at each point lies in the tangent space at that point. Each tangent space is connected with an affine connection called the Levi-Civita connection. The Christoffel symbols are the connection coefficients for the Levi-Civita connection and describe how the basis vectors of the tangent spaces change throughout a coordinate system.

Figure 43 is a high-level illustration of the technique to compute the geodesic with the equations listed above. At each point on the curve on the manifold, a derivative of the curve is taken to obtain a tangent vector that lies in the tangent space. Each tangent space is connected with an affine connection called the Levi-Civita connection [29]. This connection preserves the Riemannian metric on the manifold, is torsion-free, and allows the parallel transport of the tangent vector along the curve. The Christoffel symbols are the coefficients for the Levi-Civita connection and can be viewed as a correction to the derivative at each point to account for the change in the orientation of the basis vectors of the local coordinate system in each of the tangent spaces.

### 5.1. Numeric Computation of the Geodesic Between the Transformer Word Vector Distributions

We took a publicly available tool [30] to numerically compute the geodesic between probability distributions on a Riemannian manifold and modified it to work with the Transformer model’s word vector distributions. We used this tool to determine the relationship between the word vectors in the multi-head attention layer of the Transformer’s encoder and decoder. The tool takes samples of a D—dimensional random variable x and an M—dimensional distribution parameter a. It numerically calculates the Fisher metric $g_{ij}\left(a\right)$ from the partial derivatives of the probability distribution with respect to the parameter a. The Fisher information is computed from the logarithm of the normalized probability density function as $I\left(x,a\right)=-\log\left[f\left(x,a\right)\right]$ where $f\left(x,a\right):{\mathbb{R}}^{D}\times {\mathbb{R}}^{M}\to \mathbb{R}$. As discussed previously, the Fisher information can be interpreted as the change in KL divergence between two probability distributions as a result of infinitesimally varying the parameters $a_{i}$ and $a_{j}$ about point a and integrating over the sample space $X\in R^{D\times N}$ where N is the number of samples. The Fisher metric $g_{ij}\left(a\right)$ is numerically computed by first obtaining the elements of the Jacobian $I_{i}=\partial_{i}I$ and then using Equation (51). The Fisher metric derivative is computed as:

(56) $$\partial_{k}g_{ij}=\langle -I_{i}I_{j}I_{k}+I_{i}I_{kj}+I_{j}I_{ki}\rangle$$

The Christoffel symbols are computed according to the following expression:

(57) $$\Gamma_{ij}^{l}=\frac{1}{2}g^{lk}\left(\partial_{i}g_{kj}+\partial_{j}g_{ji}-\partial_{k}g_{ij}\right)=g^{lk}\langle I_{ij}I_{k}-\frac{1}{2}I_{i}I_{j}I_{k}\rangle$$

We used the Einstein summation notation in the above expression and this expression is the same as (55) that we had previously discussed. The term $g^{lk}$ is the inverse matrix of the term $g_{lk}$ in (55). Also, the angled brackets imply the expectation operation with respect to the probability distribution $f\left(x,a\right)$. 

The following expression is used to compute the geodesic:

(58) $${\ddot{a}}^{l}=-\Gamma_{ij}^{l}{\dot{a}}^{i}{\dot{a}}^{j}$$

where ${\dot{a}}^{i}=\frac{da^{i}}{dt}$ is the i^th^ component of a with respect to the affine parameter of the geodesic path governed by the parameter t. The solution of the geodesic expression is obtained using numeric differentiation.

### 5.2. Results of the Numeric Computation of the Geodesic Between the Transformer Word Vector Distributions of the Multihead Attention Layer of the Encoder and Decoder

**Figure 44:**
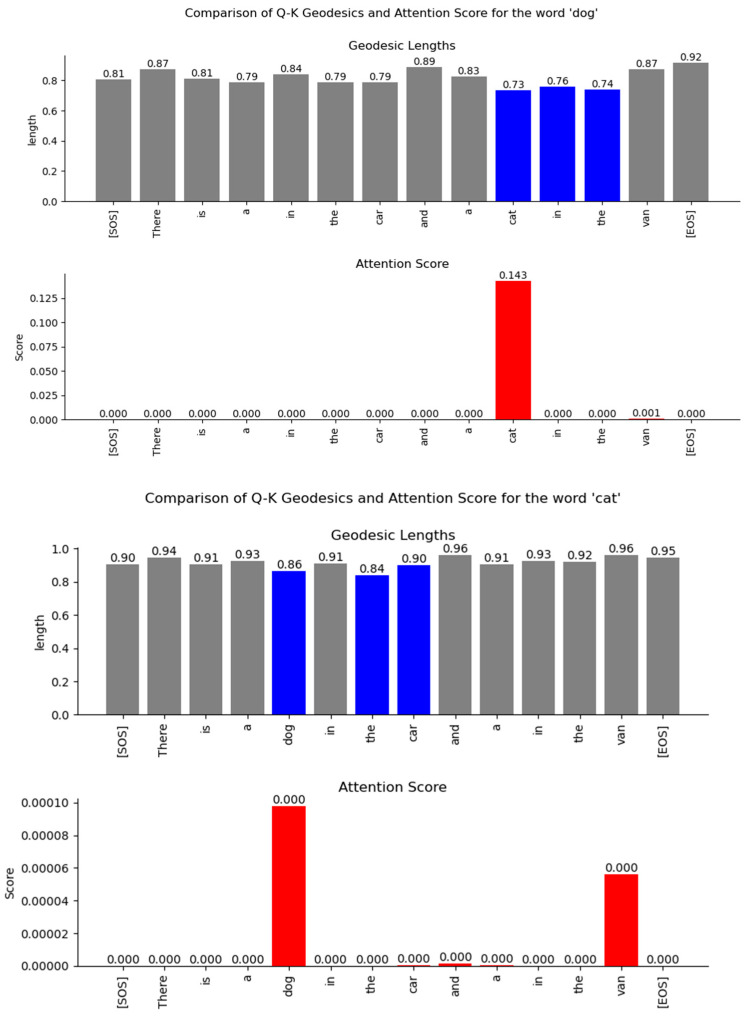
Comparison of the geodesic lengths between the Encoder’s word vector distribution and the attention scores for specific words. The comparison is shown for layer 0, attention head 0. The smaller the value of the geodesics in the high-dimensional Riemann manifold, the stronger the relationship between the words. The bars in blue mark the lowest three geodesic values, while the bars in red highlight the highest three attention scores. The relationships inferred from the length of the geodesics are similar to the attention scores. The geodesic lengths expose additional relationships between the words that are not visible in the attention score plots.

The results of the numeric computation of the geodesic between the various word vector distributions for the Q and K heads of the encoder’s multi-head attention layer are shown in Figure 44. The relationship between the words can be inferred from the length of the geodesic between the vector distributions of the words on the Riemannian manifold. The smaller the length of the geodesic in the Riemannian manifold, the stronger the relationship between the word vectors. The lengths of the geodesics compared with the attention scores show a similar trend. The geodesics expose additional weaker relationships between words that are not visible in the attention score plots.

**Figure 45:**
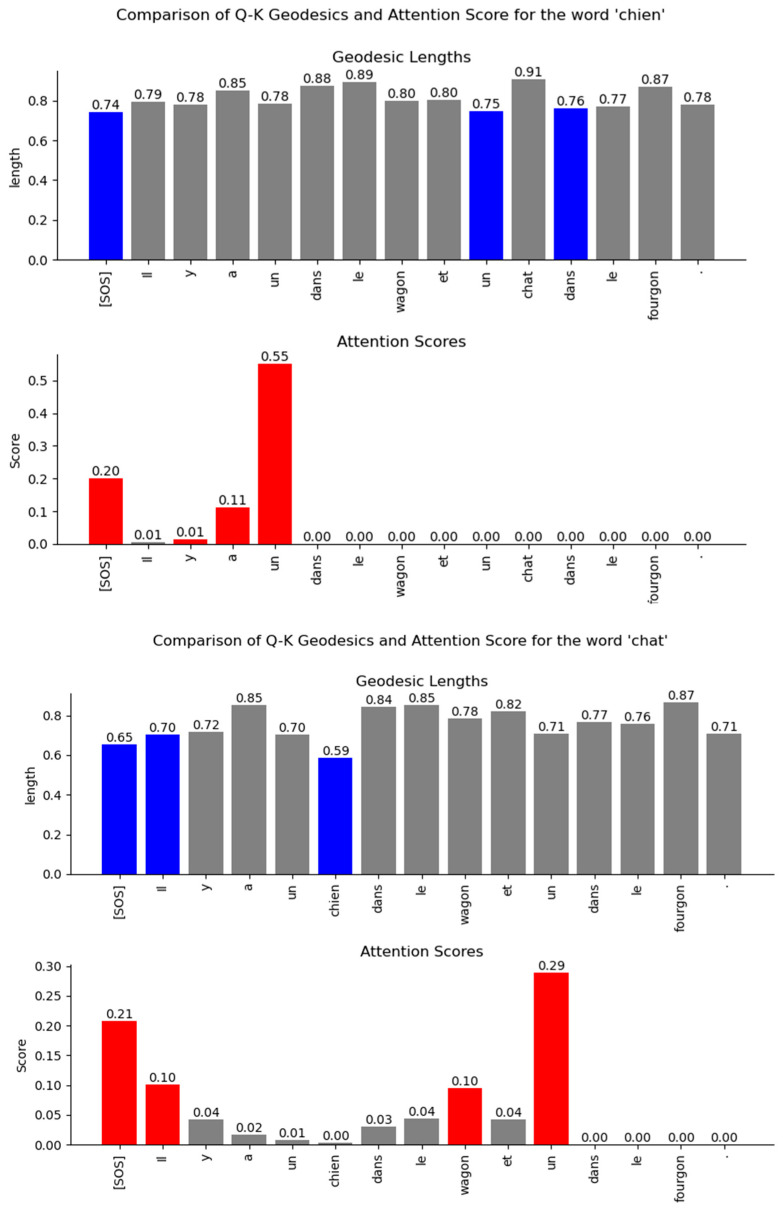
Comparison of the geodesic lengths between the Decoder’s word vector distribution and the attention scores. The comparison is shown for layer 0, attention head 0. The smaller the value of the geodesics in the high-dimensional Riemann manifold, the stronger the relationship between the words. The bars in blue mark the lowest three geodesic values, while the bars in red highlight the highest three attention scores. The relationships inferred from the length of the geodesics are similar to the attention scores. The geodesic lengths expose additional relationships between the words that are not visible in the attention score plots.

The geodesics of the word vector distributions of the Q-K heads of the Decoder’s attention layer are compared in Figure 45. The relationship between the words inferred by the length of the geodesics is similar to the attention scores. However, the geodesic lengths expose additional weaker links that are not visible in the attention score plots.

## 6. Discussion

The Information-Theoretical analysis of the Transformer model trained as a language translator provided several insights into the encoding of the relationship between input words of a sentence in a high-dimensional vector space. The tokenized words are mapped to high-dimensional vectors by the Word Embedding layer of the encoder and decoder in such a way that the distribution of the vector coordinates has very low Mutual Information with the encoded vector of a different word. Our analysis showed that the neural network in the Word Embedding layer needs the Transformer model to be of very high dimension to learn weights that can map individual word tokens to mutually independent vectors. The rows of the Positional Encoding matrix have relatively high Mutual Information even when the rows are separated by 20 indices. This ensures that positional relationships of words separated by 20 positions can be encoded by the Positional Encoding layer. The Mutual Information between the rows of the Positional Encoding matrix tapers off gradually with position difference. The effect of the geometric progression of the wavelengths of the sinusoids used in the Positional Encoding matrix is visible in the plot of the Mutual Information of this matrix.

The Query, Key, and Value input to the Encoder is mapped to a different vector space by the Projection matrices. These matrices are not idempotent, and therefore the projection of the input vectors does not lie in the column space spanned by the matrices. These matrices also don’t have any real eigenvalues. Consequently, the projections of the input to the matrix subspace are not orthogonal. The Projection matrices are of full rank and therefore have orthogonal columns. We viewed the projection as an Information channel and estimated the conditional PDF of the output given the discrete input symbols. This conditional PDF was used in the Blahut-Arimoto algorithm to compute the channel capacity of the Projection channel. The capacity of this channel was close to 0 bits, which is typical of a generative system. In a generative system, the output is mapped to a different vector space compared to the input and does not retain any part of the input. Information still flows through the Projection Information Channel, but by re-mapping the input. Since the inference is done on the transformed inputs, the Transformer assumes that the projection is a sufficient statistic of the parameters of the input. Although the Mutual Information between the input and output of the Projection Information Channel was close to 0, the joint and marginal entropies of the input and output were relatively high. We also computed the capacity of the feedforward layer, which lies at the boundary between multiple sublayers of the encoder and the decoder. We found that the capacity of this layer is also very low, implying that the output of every sublayer is mapped to a different vector space at the boundary between the layers.

For the Information-theoretic analysis of the Transformer, we had to consider the probability distribution of the coordinates of the high-dimensional vectors inside the Transformer layers. We considered the distribution of the coordinates using two different perspectives, each of which provided useful insight into the encoding of the relationship between words. One perspective considered the coordinates of the vectors to be outcomes of a single random variable. This perspective only operated on the coordinate values without regard to the dimension of the vectors. Another perspective borrowed principles from statistical mechanics to generate a Boltzmann distribution where each dimension was considered as a microstate, and the coordinate values corresponded to the energy of the microstate. The Boltzmann distribution assigned probability mass to each dimension based on the energy level of the dimension. The highlight of our study is that we illustrated how to view the encoded high-dimensional vectors in a two-dimensional Information Theoretic plane. We plotted the vector coordinate distribution on a Mutual Information vs. Wasserstein distance plane and a Bhattacharya coefficient vs. Jensen-Shannon divergence plane to visualize the relationship encoded in the high-dimensional vectors. Since our measures were based on the probability distributions, they contained all order statistics of the underlying vectors and exposed relationships not visible using attention scores.

Our Information plane view of the encoded vectors showed that during training, the Projection matrices of the Transformer learns coefficients that statistically separate the coordinate distribution of the word vectors across multiple attention heads. This allows the Transformer to learn different latent parameters of a word in different attention heads. The learning performance of the Transformer depends on the ability to statistically separate these distributions as much as possible across the multiple attention heads. We employed a statistical version of the Spectral Clustering algorithm to determine if the points in the Information Plane could be grouped into clusters with different statistics. This exercise revealed that most of the coordinate distribution in the Information Plane had very low Mutual Information between the distribution of other attention heads, yet the coordinate distribution distance was relatively close.

The Boltzmann distribution of the Projection layer inputs, which are outputs of the decoder block, showed a single peak. This resulted in a high confidence probability when the output of the Projection layer was passed through a softmax operation to select the next likely decoded word.

To illustrate how to troubleshoot performance issues within the Transformer layers, we trained a lower dimension model and analyzed the vector coordinate distribution. We used the same information-theoretical measures to compare this impaired model with the original model. Our analysis showed that with a lower-dimensional model, the Word Embedding layer is unable to learn weights to generate mutually independent embedded vectors from the input tokens. We viewed the distribution of the vector coordinates on the Mutual Information vs. Wasserstein distance plane and the Bhattacharya coefficient vs. the Jensen-Shannon divergence plane. These views showed that the impaired Transformer model is unable to separate the vector coordinate distribution across the attention heads as much as the original model. These Information plane views are an effective tool to troubleshoot performance problems in any layer of the Transformer. The Boltzmann distribution of the Projection layer input (i.e., decoder block output) showed multiple peaks with the impaired model compared to the single peak of the original model. This method of computing the word vector distribution can serve as another tool to troubleshoot the decoder performance separately from the encoder.

The difficulty of viewing the relationships between words encoded in high-dimensional vector space can be mitigated by considering the probability distribution of the word vectors as points on a Riemannian manifold. Using techniques from Information Geometry, we can compute the shortest distance or the geodesic between the points on the manifold. The Fisher information metric is the preferred metric for performing vector calculus on the tangent space of the Riemannian manifold. On a Riemannian manifold, we cannot assign position vectors to determine the distance between points. Instead, we take the derivatives at each point along the curve that connects two points and determine the tangent vector at each point. The tangent vector lies in the tangent space at a specific point on the manifold. We use a homeomorphic mapping to local coordinates in each of the tangent spaces. Each tangent space is connected with an affine connection called the Levi-Civita connection. This connection preserves the Riemannian metric on the manifold, is torsion-free, and allows the parallel transport of the tangent vector along the curve. The Christoffel symbols are the coefficients for the Levi-Civita connection and can be viewed as a correction to the derivative at each point to account for the change in the orientation of the basis vectors of the local coordinate system in each of the tangent spaces. The Fisher metric on the Riemannian manifold allows vector operations like addition and dot-product. With this metric, we were able to determine the length of each infinitesimally small tangent vector as it was parallel-transported along the line that connects two probability distribution points. The geodesic equation allowed us to numerically compute this path between two points on the Riemannian manifold. The relationships inferred from the length of the geodesic between the Transformer word vectors showed similarities to the attention scores. The geodesic lengths also exposed additional weaker relationships between words that were not apparent from the attention scores.

## 7. Conclusions

Using Information Theory, we visualized the relationship between input words encoded as high-dimensional vectors by the Transformer model. Since information theoretical analysis is based on probability distributions, it uses all order statistics of the underlying data and exposes relationships between words that were not visible to the attention scores. We analyzed the characteristics of the projection matrices and found that these matrices are not idempotent. Also, they do not project orthogonally to the space spanned by the column vectors of the matrix. We viewed the projection operation as information channels and sent discrete symbols through the channel to determine the conditional probability of the output given the input. From this, we computed the channel information capacity using the Blahut-Arimoto algorithm. We found that the capacity of the Projection information channel was very low, which is the signature of a generative model. Our method of viewing the high-dimensional vector distributions on an Information Plane of Mutual Information vs. Wasserstein distance and Bhattacharya coefficient vs. Jensen-Shannon divergence provided more insight into the relationship of the encoded word vectors compared to the attention scores. Using this Information Plane, we were able to show how to troubleshoot learning issues within the Transformer layers.

We also analyzed the Transformer word vectors encoded in a high-dimensional vector space using Information Geometry. We considered the distribution of these word vectors as points on a Riemannian manifold. To determine the relationship between the words, we numerically computed the shortest curve (geodesic) between the points on this manifold. To do this, we computed the tangent vectors at each point on the curve on the manifold by taking the derivative at that point. These tangent vectors lie on a vector space where vector calculus can be performed using the Fisher metric of the Riemannian manifold. Each of the tangent spaces is connected with an affine connection called the Levi-Civita connection, and the coefficients of this connection are known as the Christoffel symbols. These symbols serve as a correction term to the derivative at each point to account for the change in the orientation of the basis vectors of the local coordinate system in each of the tangent spaces. Our numerical computation of the geodesic lengths between word distributions using the geodesic equation enabled us to infer relationships between words that were encoded in high-dimensional space. These relationships matched the attention scores but also exposed additional relationships between words that were not apparent from the attention scores.

An Information-theoretic view of the Transformer model enables engineers to understand the non-linear relationship between the words encoded by the Transformer in a high-dimensional vector space. Our contribution in this paper helps with troubleshooting learning problems in the layers of the Transformer model by providing the ability to view the relationship between words in a high-dimensional vector space.

## Data Availability

The original contributions presented in this study are included in the article. Further inquiries can be directed to the corresponding author. No new data were created or analyzed in this study. Data sharing is not applicable to this article.

## Abbreviations

The following abbreviations are used in this manuscript:

MI	Mutual Information
PMF	Probability Mass Function
PDF	Probability Density Function
CNN	Convolutional Neural Network
KLD	Kullback-Liebler divergence
JSD	Jensen-Shannon divergence
KSG	Kraskov-Stögbauer-Grassberger
KDE	Kernel Density Estimate
MINE	Mutual Information Neural Estimator
ReLU	Rectified Linear Unit
MGF	Moment Generating Function
